# Effects of Diets High in Unsaturated Fatty Acids on Socially Induced Stress Responses in Guinea Pigs

**DOI:** 10.1371/journal.pone.0116292

**Published:** 2014-12-31

**Authors:** Matthias Nemeth, Eva Millesi, Karl-Heinz Wagner, Bernard Wallner

**Affiliations:** 1 Department of Behavioural Biology, University of Vienna, Vienna, Austria; 2 Department of Nutritional Science, University of Vienna, Vienna, Austria; 3 Cognitive Science Research Platform, University of Vienna, Vienna, Austria; Max Planck Institute of Psychiatry, Germany

## Abstract

Unsaturated fatty acids (UFAs), such as omega-3 and omega-6 poly- and omega-9 monounsaturated fatty acids are important nutrients and major components of neuronal cell membranes. They play a major role in modulating brain functions and physiology and may therefore diminish behavioral and physiological stress reactions in corroboration with decreased cortisol concentrations. Functionally, cortisol itself can modulate several behaviors and also the fatty acid metabolism in the long term. But only little is known about the behavioral and physiological influences of dietary UFAs in a social group, where individuals are regularly exposed to stressful situations. Therefore, the aim of this study was to determine the effects of dietary UFAs on saliva cortisol concentrations and behavioral responses in socially confronted guinea pigs. Three groups of animals were additionally supplemented with 500 mg chia seeds (high in omega-3), walnuts (high in omega-6), or peanuts (high in omega-9) per kg bodyweight each day and compared to a control group. During social confrontation saliva cortisol concentrations significantly increased in all groups, which was accompanied by a loss in bodyweight. However, cortisol levels remained lower in the chia and walnut groups compared to controls. Additionally, the walnut group displayed significantly increased locomotion, while no differences between groups were detected in socio-positive, sexual, or aggressive behaviors. Total plasma omega-3, omega-6, and omega-9 fatty acids were significantly increased in the corresponding groups, due to the dietary supplementations. However, a significant decrease in plasma omega-3 and an increase in plasma n-6 fatty acids were detected in the chia group when comparing the measurements before and after social confrontation. We conclude that both omega-3 and omega-6 polyunsaturated fatty acids can diminish behavioral and physiological stress responses to the social environment, enabling individuals to cope with social stressors, but at the expense of plasma derived omega-3 fatty acids.

## Introduction

Several nutrients are known to play an important role in maintaining and modulating neurobiological and physiological mechanisms in relation to behavioral expression rates [1], [2], [3]. Especially unsaturated fatty acids (UFAs) are important components of cell membrane phospholipids with highest concentrations in the central nervous system. Physiologically, these fatty acids are crucial for a healthy cephalogenesis and of major importance for maintaining body homeostasis [4], [5]. UFAs, such as omega-3 (n-3) and omega-6 (n-6) polyunsaturated fatty acids (PUFAs) and omega-9 (n-9) monounsaturated fatty acids (MUFAs), can either not be synthesized *de novo* or insufficiently in most mammalian species. The biologically most relevant PUFAs eicosapentaenoic acid (EPA; 20∶5 n-3), docosahexaenoic acid (DHA; 22∶6 n-3), and arachidonic acid (AA; 20∶4 n-6) are known as long-chain PUFAs (LC-PUFAs) and derive mainly from their dietary precursors α-linolenic acid (ALA; 18∶3 n-3) and linoleic acid (LA; 18∶2 n-6) [6], [7]. The LC- PUFAs and oleic acid (OA; 18∶1 n-9) are the most frequent fatty acids in neuromembrane phospholipids [8] and generally influence the biochemical and functional properties of cell membranes in different tissues [9]. Although the conversion of ALA and LA to the respective LC-PUFAs is not very efficient, adequate dietary intakes of these precursors result in a sufficient metabolic synthesis of LC-PUFAs for a normal neuronal development and also counteract the natural age-related loss in LC-PUFA content in the brain [10]. However, dietary modifications in any of these UFAs result in pronounced changes in an organism’s fatty acid status [11], [12], which may elicit behavioral and physiological changes.

Dietary UFAs can modulate a wide range of behaviors, including cognition, anxiety, depression, locomotion, and aggressiveness, as shown in humans and rodents [13], [14]. Varying concentrations seem to be directly related to changes in social integrity of individuals, but especially n-3 PUFAs play an important role in preventing violent and aggressive behaviors. In rats, dogs, and humans higher levels of violence and aggressiveness towards other individuals have been shown due to decreased plasma levels of ALA and DHA [15], [16], [17]. A high PUFA-diet in general may further increase locomotion, as determined in mice subjected to an open field [18]. In this context the role of MUFAs is not well documented, but a few studies reveal similar behavioral influences to the mentioned PUFAs [19], [20]. However, the n-6:n-3 ratio might constitute a critical factor for these effects. Diets low in n-3 and high in n-6 fatty acids, resulting in an elevated n-6:n-3 ratio, caused increased aggressive behavior in rats and mice, shown in classical resident-intruder tests [18], [21]. A ratio of approximately 4∶1 is suggested to have strongest effects on brain-related physiological and behavioral mechanisms and may for example reduce physiological stress responses and diminish the negative impact of stress on cognition [22]. Such UFA-related effects result from functional changes in the serotonergic system [23] and the hypothalamic-pituitary-adrenal (HPA) axis [24]. Both systems are known to be involved in the modulation of impulsive aggression and mood in relation to physiological stress responses [25], [26], [27].

The HPA-axis is generally of main importance to maintain the body homeostasis of organisms via coping with different kinds of stressors [28], [29]. An increased activity of the HPA-axis results in elevated secretion rates of the glucocorticoid cortisol, which is involved in a wide range of physiological and metabolic actions such as energy mobilization [30] and fatty acid metabolism [31]. Elevated cortisol concentrations may eventually affect the fatty acid profile as well, revealed for example by decreased plasma n-3 and MUFA concentrations due to chronic social stress in monkeys [32]. However, studies in humans and rats documented modulatory effects of dietary UFAs on the HPA-axis, resulting in decreased glucocorticoid concentrations [33], [34], [35]. According to this outcome, the negative consequences of prolonged physiological stress, such as depressive-like behaviors and cognitive deficits, are known to be counteracted by n-3 fatty acids [34], [36]. These stress-modulating effects of UFAs may be of increasing relevance since chronically increased glucocorticoid concentrations can evoke several diseases or metabolic disorders [31], [37].

Despite the above-mentioned effects of dietary UFAs on single behavior types and stress regulation, only little is known about their effects on long-term behavioral interactions and physiology in socially living animals. Kaplan et al. studied such long-term effects of high- and low-fat diets in social-living macaques and found decreased aggression and increased social integration in the high-fat group in relation to elevated neuropeptide and neurotransmitter concentrations [38], [39]. However, these studies lack analyses of specific fatty acids. But it has to be pointed out that on one hand social living per se acts as a source of physiological stress caused by competition over resources, while on the other hand socio-positive interactions can buffer these stressors [40]. Therefore, the aim of the present study was to investigate the influences of diets high in n-3, n-6, or n-9 fatty acids during a social setting in guinea pigs (*Cavia aperea f. porcellus*) where males compete for females, to monitor behavior and stress physiology in both sexes in a prolonged social context. Guinea pigs exhibit a complex social system, including harems and strict hierarchies [41], [42], and their behavioral and physiological responses to different social and environmental conditions are well studied [43], [44]. Therefore, this species is a suitable model for studying UFA-related effects on social behavior and physiological responses.

## Methods

The study was performed in accordance with the Austrian laws for animal experiments and animal keeping and permitted by the ethics committee of the faculty of life sciences, University of Vienna (2014-005), and the Austrian Federal Ministry of Science and Research (BMWF-66.006/0024-II/3b/2013).

### Animal maintenance

In total 80 domestic guinea pigs (*Cavia aperea f. porcellus*; 40 males and 40 females) were used for this study, aged 21.6±12.6 (mean ± SD) months and 781.2 g ±192.1 (mean ± SD) bodyweight. The animals were descendants of a heterogeneous, multi-colored stock, bred at the Department of Behavioural Biology at the University of Vienna, which was newly established in 2011 with animals originating from different breeders in Austria, to ensure genetic heterogeneity. All animals were sexually intact, socially skilled, accustomed to daily contact with humans, and could be individually identified by natural fur markings.

Prior to the experimental procedure animals were kept in single-sexed groups of ten individuals, housed in environmentally enriched enclosures (3.16 m^2^ each). The floor of each enclosure was covered with standard woodchip bedding material. Commercial guinea pig diet (Altromin 3023, Altromin Spezialfutter GmbH & Co. KG, Lage, Germany) and water were available *ad libitum*, The food was daily enriched with hay and fresh vegetables. Throughout the study, a light-dark cycle of 12/12 h, with lights on at 07∶00 a.m. and 22±2°C were maintained.

### Experimental diets

Throughout the experiment, the daily provided standard food per animal consisted of 25 g guinea pig pellets (Altromin 3023, Altromin Spezialfutter GmbH & Co. KG, Lage, Germany), 25 g fresh vegetables (cucumber and carrot), and 5 g hay. The amount of food was determined in preliminary studies to reach the dietary requirements of guinea pigs. The additional UFA-supplementation consisted of three natural food sources high in UFAs and low in saturated fatty acids (SFAs): chia seeds (*Salvia hispanica*), high in the n-3 ALA (ALA: 17.8%; LA: 5.8%; OA: 2.2%; SFAs: 3.3%), walnuts (*Juglans regia*), high in the n-6 LA (ALA: 9.1%; LA: 38.1%; OA: 8.8%; SFAs: 6.1%), and peanuts (*Arachis hypogaea*), high in the n-9 OA (ALA: <0.1%; LA: 15.6%; OA: 23.8%; SFAs: 8.6%); information is based on the US Department of Agriculture National Nutrient Database (all values are in % w/w). A daily amount of 500 mg chia seeds (chia group), walnuts (walnut group), or peanuts (peanut group) per kg bodyweight were dissolved in 500 µl water and administered orally using 1 ml syringes. Animals of the control group received 1 ml water per kg bodyweight. The applied procedure corresponds to previous studies where the oral administration of walnuts or purified DHA has successfully been proved in rats [45], [46].

The daily provided and supplemented foods resulted in the following percentages (% w/w) of specific fatty acids on the total food per group: chia group: ALA: 0.25%, LA: 0.64%, OA: 0.26%, SFAs: 0.23%; walnut group: ALA: 0.17%, LA: 0.96%, OA: 0.32%, SFAs: 0.26%; peanut group: ALA: 0.08%, LA: 0.74%, OA: 0.47%, SFAs: 0.28%; control group: ALA: 0.08%, LA: 0.58%, OA: 0.23%, SFAs: 0.20%. All other nutrients, including specific carbohydrates, amino acids, vitamins, and minerals, did not differ substantially between the group-specific diets (calculations based on the manufacturer’s information for the Altromin 3023 guinea pig pellets and the US Department of Agriculture National Nutrient Database; see S1 Table).

### Experimental design

Animals were randomly allocated to one of the four different experimental groups (10 males and 10 females per group). The resulting group compositions were subsequently controlled in their baseline cortisol concentrations, to exclude possible pre-experimental physiological and hierarchical influences caused by the group housing. Also the average age of the groups and sexes was controlled regarding possible differences, and revealed that they were matched to an accuracy of ±3.5 months.

The experimental procedure started with 20 days of isolation, where all animals were transferred from their single-sexed groups to single cages (100×60×45 cm). The floor of each cage was covered with woodchip bedding material and a shelter was provided. This period was followed by a three-day social confrontation test (days 21–23), which was developed in guinea pigs to monitor the animals’ behavior in a social environment [47], and subsequently by another day of isolation (day 24). Feeding procedures throughout isolation were always carried out at 11∶00 a.m. After the experiment, animals were reintroduced into their single-sexed groups. A light-dark cycle of 12/12 h, with lights on at 07∶00 a.m. and 22±2°C were maintained.

During the social confrontation test, one male and one female of each of the four groups (in total eight animals) were transferred from their cages to a squared arena (2.56 m^2^), built of wooden panels. Due to the total number of 80 animals tested in the experiment, 10 arenas were used simultaneously. The floor of each arena was again covered with woodchip bedding material, but no shelters or environmental enrichments were provided. Immediately after the transfer on day 21, social interactions were recorded for 30 minutes. These recordings were repeated on days 22 and 23. All recordings were carried out at 10∶00 a.m. using digital cameras which were located on the ceiling above the arenas. After the video recordings on day 23 all animals were returned to their cages. The animals were therefore kept in the arenas for 48 hours, except for one hour on days 21 and 22 (from 11∶00 a.m. to 12∶00 p.m.), where animals were returned to their single cages for feeding procedures. As animals ingested most of the provided food during these short periods, the remaining food was provided afterwards in the arenas containing the corresponding animals. Water was always available *ad libitum*. The animal composition of the arenas remained the same throughout the whole social confrontation test, with all animals of each single arena being present at the same time. All animals were weighed on the last day of the 20-day isolation period, on each day of the social confrontation test, and on the subsequent single day of isolation (days 20–24). Directly after weighing, saliva samples were collected, resulting in five samples per animal, to analyze saliva cortisol concentrations. The whole procedure of weighing and saliva sampling lasted a maximum time of 2 minutes per animal and was always carried out approximately at 11∶00 a.m., before UFA supplementation and feeding. Prior to social confrontation (day 20) and on the day afterwards (day 24), blood samples were collected at 12∶00 p.m., for plasma fatty acid analysis. The measurements of bodyweight and saliva cortisol concentrations during isolation, prior to and after social confrontation (days 20 and 24), represent baseline values for the non-social environment. Plasma fatty acids determined on the same days serve as marker for the general fatty acid status [48], [49].

### Sample collection

For saliva collection, a standard cotton bud was inserted into the animals’ cheek pouch for approximately 1 minute. This non-invasive method was developed in guinea pigs and reliably reflects the circulating cortisol concentrations in plasma [50], [51]. After centrifugation (14000 rpm, 17968×g, 10 minutes) samples were stored at −20°C until further analysis.

Blood samples were collected by punctuating marginal ear veins with sterile lancets and approximately 300 µl of the flowing blood was collected in heparinized micropipettes [52]. Plasma was separated by centrifugation (14000 rpm, 17968×g, 10 minutes) and stored at −20°C until further analysis.

### Saliva cortisol analysis

Saliva samples were diluted 1∶40 after thawing and cortisol concentrations measured in 10 µl inputs by biotin-strepdavidin enzyme-linked immunoassays, as described by Palme and Möstl [53], [54], using a specific antibody for cortisol (University of Veterinary Medicine, Vienna, Austria). All samples were run in duplicates. Cross-reactions with relevant steroids were: 4-pregnene-11β,21-diol-3,20-dione 6.2%; 4-pregnene-11β,17α,21-triol-3,20, dione 100%; 5α-pregnane-11β,17α,21-triol-3,20, dione 4.6%; 5α-pregnane-3α,11β,17α,21-tetrol-20-one 0.8%; 5β-pregnane 3α,11β,17α,21-tetrol-20-one 0.1%; all other steroids cross-reacted <0.01%. Intra- and interassay coefficients of variance were 8.1% and 13.4%.

### Plasma fatty acid analysis

Determination of plasma fatty acids was carried out using gas chromatography, based on the method of Wagner et al. [55]. Fatty acids were transesterificated by adding 1 ml methanolic NaOH, containing butylated hydroxytoluene (BHT) to prevent oxidation, to 100 µl plasma. Thereafter, 1 ml 14% boran-triflourid-methanol (BF_3_) was added to obtain fatty acid methyl esters (FAMES). After FAMES were extracted into 500 µl hexane four times, they were vaporized and re-dissolved in hexane. Using an Auto-System-Gaschromatograph (Perkin Elmer, USA) with flame ionization detector (FID), FAMES were separated by a Rtx-2330 30 m × 0.25 mm i.d. silica column. 1 µl of prepared samples were injected at a temperature of 250°C and detected at 270°C; helium was used as carrier gas. Identification of fatty acids was done by a 37 component FAME Mix Standard (Supelco, Bellafonte, USA). For peak integration, TotatChrome Workstation 6.3.0 (PE Nelson, Perkin Elmer, USA) was used. For further analysis, single fatty acids were summed up to calculate the total percentages of n-3, n-6, n-9, SFAs, MUFAs, and PUFAs, respectively the ratios of n-6: n-3 fatty acids (n-6:n-3 ratio), monounsaturated: saturated fatty acids (M:S ratio), and polyunsaturated: saturated fatty acids (P:S ratio).

### Behavioral measures

Behavior was recorded three times throughout the social confrontation test and quantified using The Observer XT 10 software package (Version 10.5, Noldus, Wageningen, the Netherlands). All types of behavior were measured by applying a continuous/all occurrence sampling method for each individual [56]. Behavioral categories and their definitions followed Rood [57]: (1) Locomotion: walking, running, jumping. (2) Socio-positive behavior: side by side (huddling), social grooming, nose-nose contact. (3) Agonistic behavior: displacement, chasing, fighting, biting, teeth chatter, head-thrust, stand-threat, kick-back, riding (male vs. male), rumba-rumble (male vs. male). (4) Sexual behavior: marking, naso-anal contact, chin-rump follow, rumba-rumble (male vs. female), riding (male vs. female), copulation. Locomotor behaviors were measured in durations, all other types of behavior in frequencies.

### Statistical analysis

As all relevant measurements were carried out on days 20–24 (see above), for simplification these days are for all statistics referred to as ‘preSoc’ (day 20, the day prior to social confrontation), ‘Soc1’, ‘Soc2’, and ‘Soc3’ (days 21, day 22, and day 23, the three days of social confrontation), and ‘postSoc’ (day 24, the day after social confrontation).

Statistics were carried out using R 3.0.0 [58]. Linear mixed effect models (LME’s, library ‘nlme’ [59]) with type-III sum of squares were used to control for differences in displayed behaviors (locomotion, socio-positive behavior, agonistic behavior, and sexual behavior), saliva cortisol concentrations, bodyweight, and plasma fatty acids between groups, sexes, and days, considering the repeated measurements. Therefore the factorial predictors ‘group’ (chia, walnut, peanut, control), ‘sex’ (male, female), and ‘day’ (dependent on the response variable; behaviors: Soc1, Soc2, Soc3; saliva cortisol and bodyweight: preSoc, Soc1, Soc2, Soc3, postSoc; plasma fatty acids: preSoc, postSoc) were included as fixed factors in each model, and single individuals as random effect (for the repeated measurements). Starting with three-way interactions (full models), models were fitted (stepwise deletion of non-significant terms) based on the Akaike information criterion (AIC). Significant terms of the fitted models were further analyzed by applying post-hoc interaction analyses (library ‘phia’ [60]) with bonferroni corrections and if possible significant effects on saliva cortisol concentrations and bodyweight were based on the days ‘preSoc’, ‘Soc1-3’, or ‘postSoc’ only. Post-hoc interactions analyses were also carried out to check for day-related effects within single groups and/or sexes.

Model assumptions (normal distribution, homogeneity of variance, and linearity) were checked by visual inspection of the residuals and fitted values and by applying Shapiro-Wilk and Levene tests. Some of the data had to be transformed by applying the natural logarithm or taking the second or third root, but were back-transformed for visualization of the results. Only terms and interactions which remained in the fitted models are considered in the result section; for statistics of removed terms and interactions see S2 Table. Significance was set at a level of p≤0.05. Values are means ± SEM.

## Results

### Behavioral measurements

**Figure 1:**
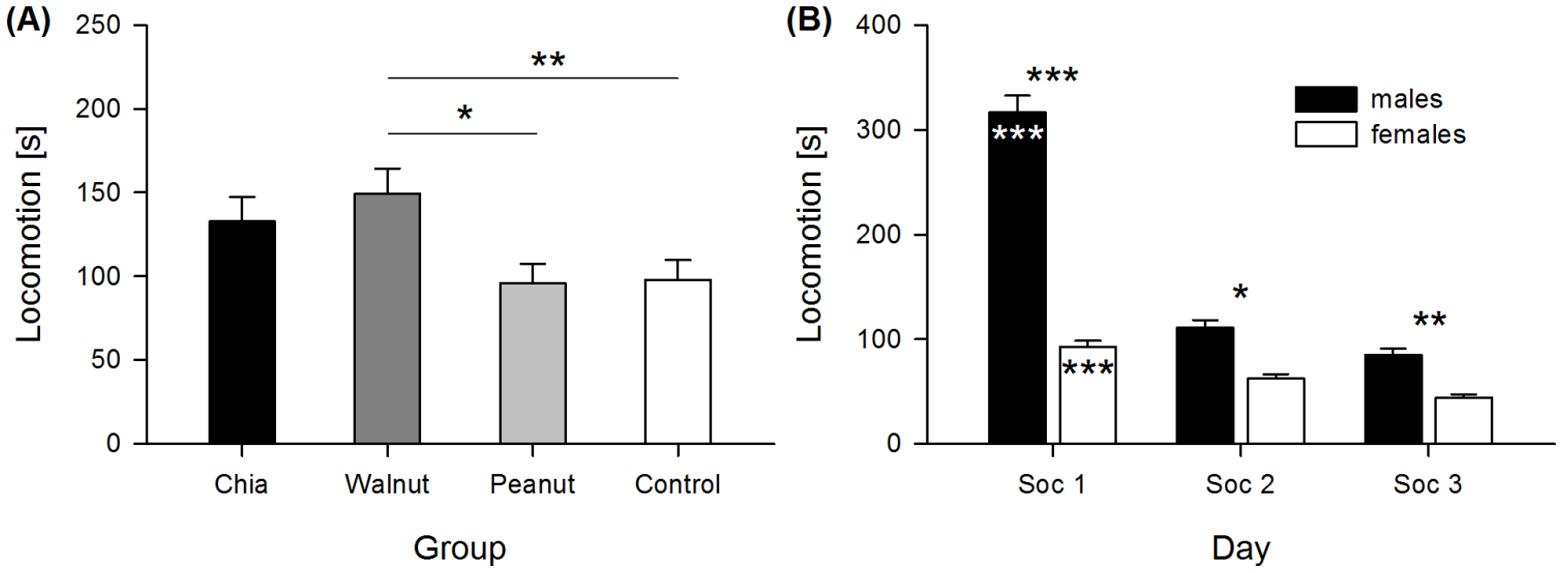
Duration of locomotion during the social confrontation test. (A) Mean duration of locomotion for the groups during the social confrontation test. (B) Duration of locomotion in males and females for each day of the social confrontation test. Asterisks within bars indicate significant changes from day to day in males and females. * p≤0.05, ** p≤0.01, *** p≤0.001.

During the social confrontation test significant differences in the duration of locomotion were detected between groups, sexes, and days (group *F*_3,74_ = 3.464, *p* = 0.020; sex *F*_1,74_ = 51.912, *p*<0.001; day *F*_2,154_ = 8.852, *p*<0.001) (Fig. 1). A significant interaction of sex and day was detected as well (sex:day *F*_2,154_ = 8.823, *p*<0.001). Individuals of the walnut group showed increased locomotion compared to the control and peanut group (walnut-control = 0.425, *χ^2^* = 7.013, *p* = 0.008; walnut-peanut = 0.404, *χ^2^* = 6.502, *p* = 0.011). The chia group also displayed slightly increased locomotion, but no further differences between groups were significant (chia-control = 0.301, *χ^2^* = 3.520, *p* = 0.061; chia-peanut = 0.280, *χ^2^* = 3.124, *p* = 0.077; chia-walnut = −0.124, *χ^2^* = 0.612, *p* = 0.434; control-peanut = −0.021, *χ^2^* = 0.017, *p* = 0.895) (Fig. 1A). Locomotion was generally higher in males than in females on each day, but the decrease during the test period was less strong in females (post-hoc analysis: males: Soc1–Soc2 = 0.990, Soc1–Soc3 = 1.201, Soc2–Soc3 = 0.211, *χ^2^* = 97.992, *p*<0.001; females: Soc1–Soc2 = 0.299, Soc1–Soc3 = 0.551, Soc2–Soc3 = 0.251, *χ^2^* = 17.704, *p*<0.001; males-females: Soc1 = 1.117, *χ^2^* = 51.912, *p*<0.001; Soc2 = 0.427, *χ^2^* = 7.594, *p* = 0.018; Soc3 = 0.468, *χ^2^* = 9.098, *p* = 0.008) (Fig. 1B).

Socio-positive behaviors did not differ between groups, sexes, or days, and also no interaction effects were detected (see S2 Table). The overall mean in the frequency of socio-positive behaviors for groups, sexes, and days was 15.92±0.94.

**Figure 2:**
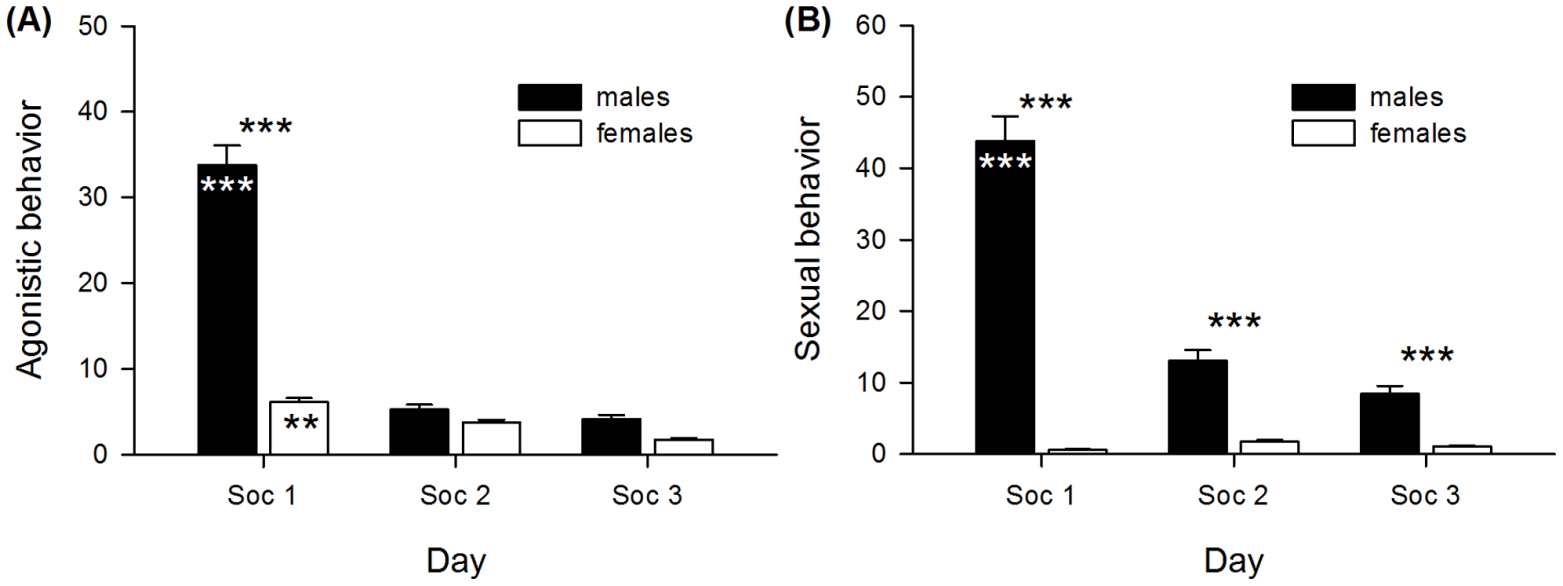
Displayed social behaviors during the social confrontation test. (A) Frequencies of agonistic behavior and (B) sexual behavior in males and females for each day of the social confrontation test. No group-related effects on displayed behaviors were detected (see text). Asterisks within bars indicate significant changes from day to day in males and females. ** p≤0.01, *** p≤0.001.

Agonistic behaviors differed between the sexes and days (sex *F*_1,77_ = 39.953, *p*<0.001; day *F*_2,154_ = 7.090, *p* = 0.001) and also the interaction of sex and day was significant (sex:day *F*_2,154_ = 12.935, *p*<0.001). Males generally displayed more agonistic behaviors than females, but also showed a very strong decline. The sex-difference was therefore only based on day Soc1 (post-hoc analysis: males: Soc1–Soc2 = 1.164, Soc1–Soc3 = 1.275, Soc2–Soc3 = 0.111, *χ^2^* = 105.660, *p*<0.001; females: Soc1–Soc2 = 0.225, Soc1–Soc3 = 0.533, Soc2–Soc3 = 0.298, *χ^2^* = 14.180, *p* = 0.002; males-females: Soc1 = 1.039, *χ^2^* = 39.953, *p*<0.001; Soc2 = 0.100, *χ^2^* = 0.368, *p* = 1.000; Soc3 = 0.287, *χ^2^* = 3.042, *p* = 0.243) (Fig. 2A).

Male sexual behaviors followed the same patterns as agonistic behaviors, whereas females displayed nearly no sexually intended behaviors at all (sex *F*_1,77_ = 129.139, *p*<0.001; day *F*_2,154_ = 2.467, *p* = 0.088; sex:day *F*_2,154_ = 27.016, *p*<0.001; post-hoc analysis: males: Soc1–Soc2 = 1.227, Soc1–Soc3 = 1.571, Soc2–Soc3 = 0.344, *χ^2^* = 74.825, *p*<0.001; females: Soc1–Soc2 = −0.429, Soc1–Soc3 = −0.224, Soc2–Soc3 = 0.205, *χ^2^* = 4.935, *p* = 0.170; males-females: Soc1 = 2.747, *χ^2^* = 129.139, *p*<0.001; Soc2 = 1.091, *χ^2^* = 20.367, *p*<0.001; Soc3 = 0.952, *χ^2^* = 15.515, *p*<0.001) (Fig. 2B).

No group-related effects, including main effects and interactions with sex or day, were detected regarding socio-positive, agonistic, or sexual behaviors (see S2 Table).

### Saliva cortisol concentrations

**Figure 3:**
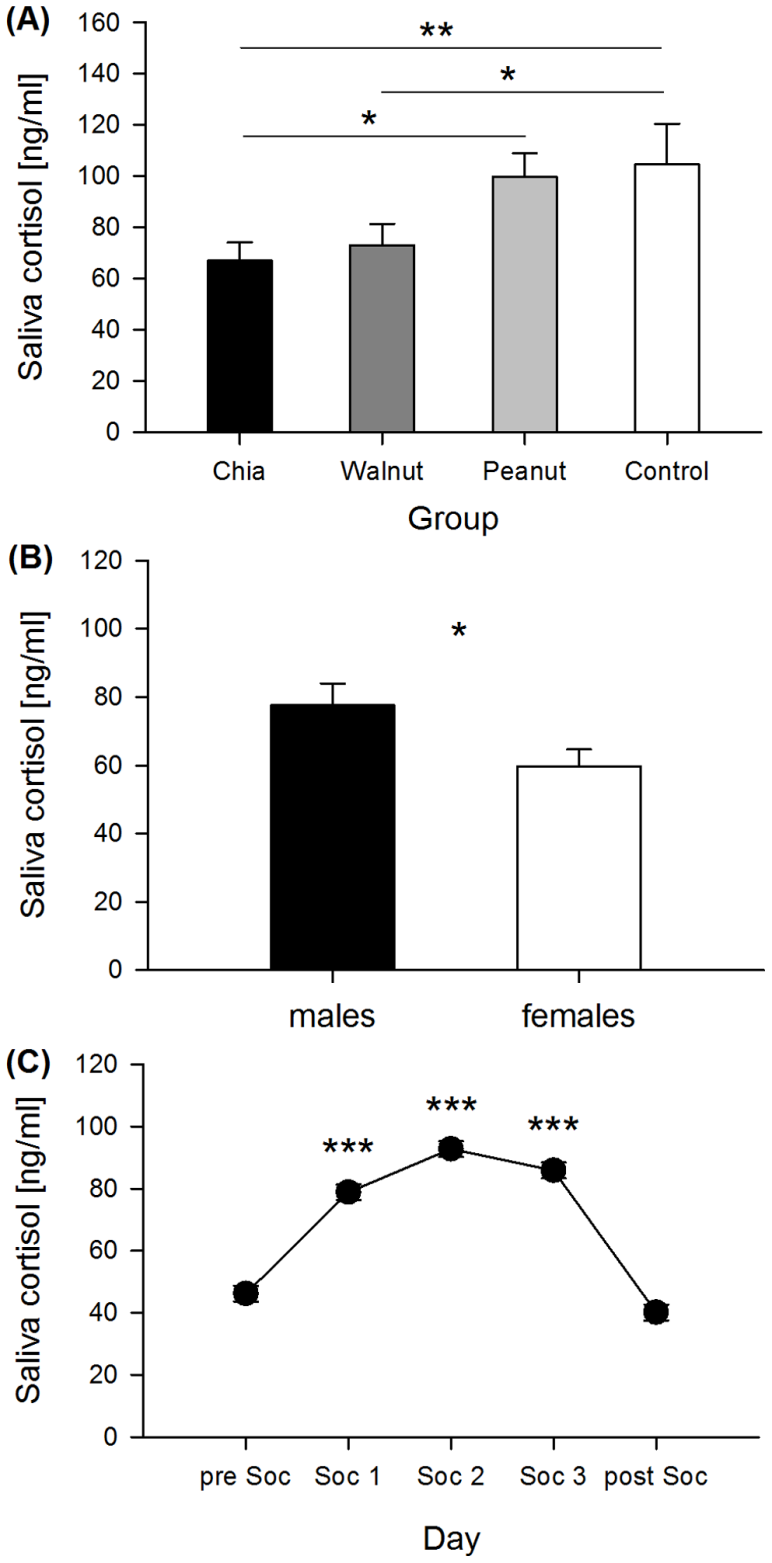
Saliva cortisol concentrations. (A) Mean saliva cortisol concentrations for the groups during the social confrontation test. (B) Mean saliva cortisol concentrations for males and females throughout the experimental procedure (before, during, and after the social confrontation test). (C) Daily change in mean saliva cortisol concentrations, including the days before the social confrontation test (preSoc), the three days of social confrontation (Soc1-3), and after the social confrontation test (postSoc), which did not differ between the groups and sexes (see text). * p≤0.05, ** p≤0.01, *** p≤0.001.

Saliva cortisol concentrations were significantly different between the groups, sexes, and days (group *F*_3,74_ = 2.963, *p* = 0.038; sex *F*_1,74_ = 5.266, *p* = 0.025; day *F*_4,312_ = 16.013, *p*<0.001) (Fig. 3). As revealed by post-hoc interaction analysis the group effect was only based on the three days of social confrontation (Soc1–Soc3). Saliva cortisol concentrations were lower in the chia group compared to the control and peanut group (chia-control = −37.171, *χ^2^* = 6.686, *p* = 0.009; chia-peanut = −32.736, *χ^2^* = 5.324, *p* = 0.021). The walnut group also showed lower concentrations compared to the control group and marginally to the peanut group (walnut-control = −31.054, *χ^2^* = 4.666, *p* = 0.031; walnut-peanut = −26.619, *χ^2^* = 3.520, *p* = 0.061). No further differences between groups were found (control-peanut = 4.435, *χ^2^* = 0.095, *p* = 0.758; chia-walnut = −6.117, *χ^2^* = 0.186, *p* = 0.666) (Fig. 3A). In contrast to the group effect, the effect of sex was present on all days (preSoc, Soc1-3, postSoc), with males consistently exhibiting higher cortisol concentrations than females (Fig. 3B). Regarding the daily changes, saliva cortisol concentrations were significantly higher throughout all three days of social confrontation (Soc1–Soc3) compared to the days preSoc and postSoc. But concentrations did not differ between preSoc and postSoc and did also not change throughout social confrontation (preSoc-Soc1 = −32.685, *χ^2^* = 14.875, *p* = 0.001; preSoc-Soc2 = −46.591, *χ^2^* = 30.223, *p*<0.001; preSoc-Soc3 = −39.700, *χ^2^* = 21.944, *p*<0.001; preSoc-postSoc = 6.031, *χ^2^* = 0.506, *p* = 1.000; Soc1–Soc2 = −13.906 *χ^2^* = 2.692, *p* = 1.000; Soc1–Soc3 = −7.015, *χ^2^* = 0.685, *p* = 1.000; Soc1–postSoc = 38.716, *χ^2^* = 20.870, *p*<0.001; Soc2–Soc3 = 6.891, *χ^2^* = 0.661, *p* = 1.000; Soc2-postSoc = 52.621, *χ^2^* = 38.554, *p*<0.001; Soc3-postSoc = 45.730, *χ^2^* = 29.117, *p*<0.001) (Fig. 3C).

### Bodyweight

**Figure 4:**
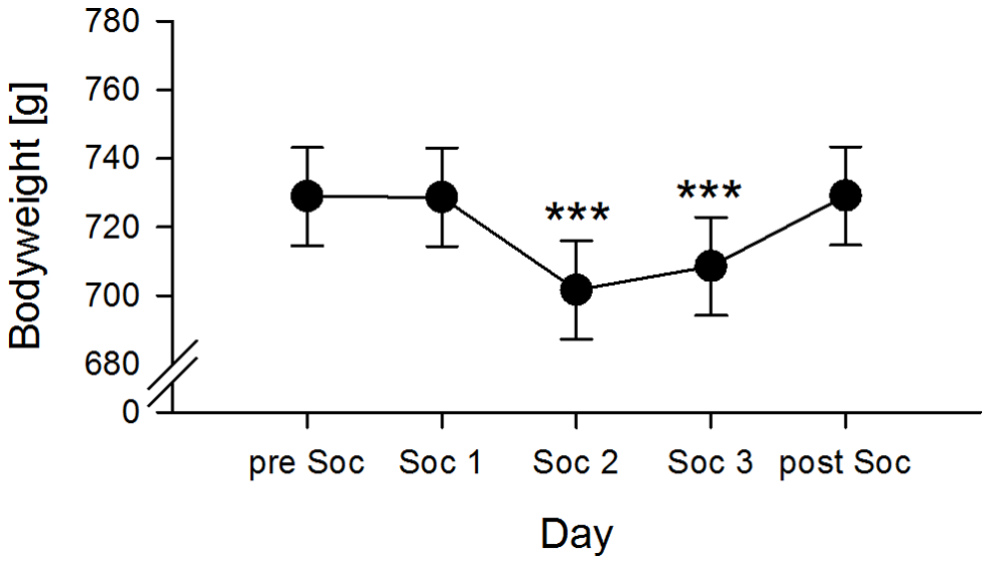
Bodyweight. Mean bodyweight for each day of the experimental procedure, including the days before the social confrontation test (preSoc), the three days of social confrontation (Soc1-3), and after the social confrontation test (postSoc), which did not differ between the groups and sexes (see text). *** p≤0.001.

The animals’ bodyweight only differed between the days (day *F*_4,312_ = 94.917, *p*<0.001), while no group- or sex-related differences were detected (see S2 Table). Animals of all groups and sexes showed a significantly decreased bodyweight on days Soc2 and Soc3 compared to all the other days (preSoc-Soc1 = 0.190, *χ^2^* = 0.010, *p* = 1.000; preSoc-Soc2 = 27.215, *χ^2^* = 202.952, *p*<0.001; preSoc-Soc3 = 20.329, *χ^2^* = 113.242, *p*<0.001; preSoc-postSoc = −0.177, *χ^2^* = 0.009, *p* = 1.000; Soc1–Soc2 = 27.025, *χ^2^* = 200.130, *p*<0.001; Soc1–Soc3 = 20.139, *χ^2^* = 111.137, *p*<0.001; Soc1–postSoc = −0.367, *χ^2^* = 0.037, *p* = 1.000; Soc2–Soc3 = −6.886, *χ^2^* = 12.993, *p* = 0.003; Soc2-postSoc = −27.392, *χ^2^* = 205.604, *p*<0.001; Soc3-postSoc = −20.506, *χ^2^* = 115.225, *p*<0.001) (Fig. 4).

### Plasma fatty acids

The dietary treatments significantly affected the percentages of different types of fatty acids in plasma (Table 1). Irrespective of groups and sexes, the total percentages of plasma n-3, n-6, and n-9 fatty acids were predominantly represented by the UFAs which were also present in the diet, namely ALA, LA, and OA (ALA: 88.21±0.74% on total n-3, LA: 93.25±0.17% on total n-6, OA: 98.19±0.17% on total n-9). The percentages of n-3 and n-6 LC-PUFAs in plasma were quite small and represented only 11.79±6.34% on total n-3 and 6.18±1.45% on total n-6.

**Table 1:** Plasma fatty acids (% on total fatty acids; mean ± SEM) comparing groups, sexes, and days (pre Soc: before the social confrontation test; post Soc: after the social confrontation test).

Fatty Acid	Day	Group				Sex		Overall mean
		Chia	Walnut	Peanut	Control	males	females	
16∶0	pre Soc	14.62±0.33	12.99±0.83	13.52±0.40	14.82±0.74	13.41±0.56^a^	14.56±0.27^b^	14.01±0.31
	post Soc	14.37±0.42	13.45±0.35	13.38±0.34	14.11±0.63	13.40±0.38^a^	14.22±0.25^b^	13.83±0.23
18∶0	pre Soc	11.70±0.32^ab^	10.92±0.51^a^	11.39±0.29^ab^	12.24±0.68^b^	12.04±0.40	11.15±0.27	11.58±0.24
	post Soc	12.02±0.39^ab^	10.83±0.44^a^	11.30±0.24^ab^	12.57±0.57^b^	12.62±0.33^a^	10.84±0.23^b^	11.69±0.22
18∶1 n-9	pre Soc	11.97±0.72^a^	12.69±0.59^a^	17.16±0.68^b^	12.48±0.29^a^	14.12±0.06	13.17±0.51	13.62±0.38
	post Soc	12.60±0.41^a^	12.55±0.76^a^	17.30±0.85^b^	12.49±0.29^a^	13.59±0.61	13.96±0.50	13.78±0.39
20∶1 n-9	pre Soc	0.12±0.02^a^	0.13±0.03^a^	0.23±0.02^b^	0.19±0.04^a^	0.19±0.02	0.15±0.02	0.17±0.01
	post Soc	0.13±0.02^a^	0.12±0.02^a^	0.21±0.01^b^	0.13±0.01^a^	0.16±0.02	0.14±0.01	0.15±0.01
18∶2 n-6	pre Soc	43.37±1.09^a^	48.05±1.14^b^	44.22±0.91^a^	44.40±0.98^a^ *	44.64±0.77	45.24±0.78	44.95±0.54***
	post Soc	44.73±1.09^a^	48.64±0.77^b^	45.06±1.05^a^	46.31±0.66^a^	45.87±0.67	46.39±0.69	46.14±0.48
20∶2 n-6	pre Soc	0.30±0.02	0.28±0.02	0.30±0.02	0.34±0.02	0.30±0.02	0.30±0.01	0.30±0.01
	post Soc	0.28±0.02	0.30±0.03	0.29±0.02	0.33±0.02	0.30±0.02	0.30±0.02	0.30±0.01
20∶4 n-6	pre Soc	2.36±0.11^ab^	2.25±0.10^a^	2.73±0.14^b^	2.65±0.17^ab^	2.43±0.10***	2.57±0.10	2.51±0.07
	post Soc	2.76±0.17^ab^	2.54±0.17^a^	2.87±0.20^b^	2.84±0.14^ab^	2.91±0.13	2.62±0.11	2.76±0.09
18∶3 n-3	pre Soc	9.58±0.60^a^***	6.20±0.30^b^	4.07±0.19^c^	5.23±0.26^b^	6.56±0.51^a^***	5.92±0.34^b^	6.23±0.30
	post Soc	7.95±0.60^a^	5.61±0.37^b^	3.97±0.19^c^	4.81±0.22^b^	5.45±0.37	5.65±0.35	5.55±0.25
20∶5 n-3	pre Soc	0.07±0.02	0.05±0.02	0.07±0.02	0.09±0.02	0.07±0.01	0.07±0.01	0.07±0.01
	post Soc	0.06±0.02	0.07±0.02	0.07±0.02	0.06±0.02	0.07±0.01	0.06±0.01	0.06±0.01
22∶5 n-3	pre Soc	0.38±0.04	0.29±0.02	0.35±0.04	0.37±0.03	0.32±0.02	0.37±0.03	0.35±0.02
	post Soc	0.41±0.04	0.33±0.03	0.38±0.06	0.37±0.03	0.37±0.03	0.38±0.03	0.38±0.02
22∶6 n-3	pre Soc	0.24±0.05	0.25±0.03	0.29±0.06	0.33±0.05	0.25±0.03	0.30±0.04	0.28±0.02
	post Soc	0.30±0.05	0.27±0.03	0.29±0.06	0.26±0.02	0.25±0.02	0.30±0.04	0.28±0.02
total n-9	pre Soc	12.12±0.72^a^	12.93±0.58^a^	17.52±0.71^b^	12.75±0.29^a^	14.40±0.58	13.39±0.52	13.88±0.39
	post Soc	12.78±0.40^a^	12.76±0.75^a^	17.63±0.86^b^	12.69±0.29^a^	13.85±0.62	14.17±0.51	14.01±0.40
total n-6	pre Soc	46.36±1.07^a^ *	50.92±1.11^b^	47.58±0.89^a^	47.75±0.99^a^ *	47.69±0.76**	48.48±0.75*	48.10±0.53***
	post Soc	48.10±1.09^a^	51.81±0.84^b^	48.54±1.18^a^	49.89±0.72^a^	49.43±0.74	49.65±0.71	49.54±0.51
total n-3	pre Soc	10.26±0.56^a^***	6.79±0.31^b^	4.78±0.24^c^	6.02±0.24^b^	7.21±0.50***	6.66±0.34	6.92±0.30
	post Soc	8.73±0.59^a^	6.28±0.38^b^	4.71±0.22^c^	5.50±0.22^b^	6.15±0.37	6.39±0.36	6.27±0.26
total SFA	pre Soc	29.70±0.77^ab^	27.27±1.12^a^	28.19±0.87^a^	31.29±0.98^b^	28.85±0.76	29.44±0.65**	29.15±0.49**
	post Soc	28.93±0.61^ab^	27.24±0.64^a^	27.27±0.48^a^	29.85±0.57^b^	28.84±0.44	27.89±0.43	28.34±0.31
total MUFA	pre Soc	13.68±0.67^a^	15.02±0.60^a^	19.45±0.68^b^	14.95±0.27^a^	16.26±0.56	15.42±0.53	15.82±0.39
	post Soc	14.25±0.42^a^	14.67±0.78^a^	19.49±0.89^b^	14.76±0.30^a^	15.59±0.62	16.08±0.53	15.84±0.40
total PUFA	pre Soc	56.62±0.94^ab^	57.71±1.10^a^	52.36±0.86^c^	53.77±0.95^bc^	54.89±0.79	55.14±0.73	55.02±0.53*
	post Soc	56.82±0.87^ab^	58.09±0.71^a^	53.24±1.10^c^	55.39±0.67^bc^	55.58±0.69	56.03±0.65	55.81±0.47
n-6: n-3	pre Soc	4.82±0.34^a^***	7.87±0.58^b^	10.46±0.62^c^	8.21±0.43^bc^ *	7.58±0.46***	8.15±0.51	7.88±0.34
	post Soc	6.17±0.65^a^	9.11±0.97^b^	10.86±0.73^c^	9.37±0.45^bc^	9.01±0.56	8.81±0.59	8.91±0.40
M: S	pre Soc	0.47±0.02^a^	0.57±0.04^a^	0.71±0.04^b^	0.49±0.02^a^	0.58±0.03	0.54±0.02	0.56±0.02*
	post Soc	0.49±0.01^a^	0.55±0.04^a^	0.72±0.03^b^	0.50±0.01^a^	0.55±0.02	0.58±0.02	0.57±0.02
P: S	pre Soc	1.94±0.08^ab^	2.20±0.13^a^	1.90±0.08^ab^	1.77±0.08^b^	1.97±0.08	1.92±0.06*	1.94±0.05*
	post Soc	1.99±0.07^ab^	2.15±0.06^a^	1.97±0.07^ab^	1.88±0.06^b^	1.95±0.05	2.03±0.05	1.99±0.03

Different superscripts indicate significant differences between groups and/or sexes on days pre Soc and post Soc (*p*≤0.05).

*p≤0.05, **p≤0.01, *p*≤0.001 comparing single groups and/or sexes on days pre Soc and post Soc.

Corresponding to the most prominent fatty acids in the diets, the percentage of plasma ALA was highest in the chia group, plasma LA was highest in the walnut group, and plasma OA highest in the peanut group. Single and total SFAs were highest in the control group. According to the elevated concentrations of single fatty acids, the chia groups also exhibited the lowest plasma n-6:n-3 ratio. As the walnut and peanut groups also showed the lowest percentages in total SFAs, the walnut group additionally exhibited the highest P:S ratio and the peanut group the highest M:S ratio in plasma. No differences were detected in the LC- PUFAs AA, EPA, or DHA (Table 1).

Comparing the measurement preSoc and postSoc revealed a decrease in plasma n-3 fatty acids (ALA, total n-3) and an increase in plasma n-6 fatty acids (LA, total n-6) in the chia group. Plasma n-6 fatty acids (LA, total n-6) also increased in the control group. This also caused an increase in the plasma n-6:n-3 ratio in the chia and control groups (Table 1).

## Discussion

The present study was designed to determine behavioral and physiological influences of different dietary sources high in UFAs, namely the n-3 ALA (highest in the chia group) the n-6 LA (highest in the walnut group), and the n-9 OA (highest in the peanut group), compared to an untreated control group in socially confronted guinea pigs. The results highlight the importance of n-3 and n-6 as dietary components for diminishing physiological and behavioral stress responses, as indicated by decreased saliva cortisol concentrations and increased locomotion. Further on, the plasma n-3 and n-6 status seemed to be affected by increased cortisol concentrations, resulting in an increase in the n-6:n-3 ratio due to the applied social stressor.

20 days of supplementation with diets high in different UFAs resulted in significant differences in the plasma fatty acid status between groups. Feeding on chia seeds, walnuts, and peanuts resulted in increased plasma n-3, n-6, and n-9 fatty acids, as well as decreased plasma SFAs compared to the non-supplemented control group. This primarily proved the successful uptake of UFAs from the diet. But findings in rats indicate that the integration of plasma fatty acids into neuronal cell membranes only takes 1–2 weeks [61] and that plasma fatty acids serve as reliable markers for the tissue fatty acid composition [49]. Therefore, we conclude that the differences in plasma fatty acids detected here are also related to differences in tissue compositions, even in the brain. Further on, we also detected differences between groups in the plasma n-6:n-3 ratio (lowest in the chia group), the P:S ratio (highest in the walnut group), and the M:S ratio (highest in the peanut group). N-6 and n-3 fatty acids share some metabolism pathways [62], while SFAs seem to have negative effects on the fatty acid and lipid metabolism [63], [64]. As a decreased n-6:n-3 ratio and increased P:S and M:S ratios were found for the supplemented groups, this further indicates the beneficial effects of the applied dietary treatments on the plasma UFA status.

The social confrontation test caused an increase in saliva cortisol concentrations in all groups, immediately after the onset of the test, and cortisol levels remained elevated throughout the testing procedure. However, cortisol concentrations remained lower in the chia and walnut groups throughout the three days of social confrontation, indicating a diminishing effect of n-3 and n-6 fatty acids on the HPA-axis reactivity under social conditions. Diets enriched in n-3, including ALA and DHA, have repeatedly been shown to decrease glucocorticoid concentrations in rats due to different stressors [34], [65]. The same effect was also found for a n-6:n-3 ratio of 4∶1 [66], a ratio that was approximately measured in the plasma of the chia group. In contrast, diets enriched only in n-6 may even facilitate glucocorticoid secretion rates [67]. As also the walnut group (remember high in n-6) showed lower saliva cortisol levels, this result seems to be in a strong contrast to the mentioned finding. However, the walnut group also showed the highest plasma PUFA level in general, which is the entirety of total n-3 and n-6 fatty acids, and even exhibited the highest P:S ratio. The decreased saliva cortisol concentrations found in the walnut group may therefore be related to the total PUFA intake and not to dietary n-6 fatty acids alone. This would indicate the importance of PUFAs in general, including dietary supplementations of both n-3 and n-6, for diminishing physiological stress reactions.

Interestingly, lower saliva cortisol concentrations throughout social confrontation seemed to be related to longer durations of locomotor activity. Although significantly more locomotion compared to control and peanut groups were only found in the walnut group (high in n-6), also the chia group (high in n-3) showed slightly increased locomotor activity. The relationship between stress perception and activity in guinea pigs was shown in other studies [44], [68], which documented tonic immobility as a behavioral stress reaction. Previous studies on the effects of UFAs showed a positive influence of ALA and DHA on locomotion and the general activity frequencies in rodents and non-human primates [69], [70], which was also related to decreased glucocorticoid concentrations [36]. The results of our study indicate that walnuts, perhaps also chia seeds, may counteract a stress-induced immobility during social confrontation, and thus the behavioral stress response. In contrast, peanuts (high in n-9) seemed to have no effects on the physiological and behavioral stress response in guinea pigs, since no differences to control animals in behavior and physiological stress reactions were found.

However, no differences between the groups were found for socio-positive, agonistic, or sexual behaviors. Previous studies in rodents and humans showed that n-3 fatty acids or lower n-6:n-3 ratios result in less aggression or violence [14], [71]. Also glucocorticoids are known to play an important role in modulating behavioral expression rates [27]. As saliva cortisol concentrations significantly increased in all groups due to social confrontation, behavioral expressions could have been affected in a negative way in all groups, although cortisol levels remained lower in the chia and walnut groups. Therefore, influences of the dietary treatments were possibly counteracted by the elevated stress-load in all groups.

Parallel to an increased HPA-axis activity in all groups during the social confrontation test, a decrease in bodyweight was detected, demonstrating a negative consequence of the long-term stress load. The HPA-axis and glucocorticoid concentrations play an important role in maintaining and modulating bodyweight [72]. In rodents, as for example in rats, increased glucocorticoid concentrations can result in a dramatic weight loss [35], [73]. Diminishing effects of n-3 fatty acids on glucocorticoid concentrations may result in a lower loss in bodyweight or even counteract this effect completely [35]. No such effects were observed in this study, as bodyweight did not differ between groups prior to, during, or after social confrontation. As saliva cortisol concentrations and bodyweight returned to pre test levels immediately after social confrontation, guinea pigs are possibly well adapted to such situations. This could especially be true in males, as their success in monopolizing females strongly depends on their conflict involvement and related cortisol concentrations [74].

Also the observed changes in plasma fatty acids are suggested to be caused by the increase in saliva cortisol concentrations. After the social confrontation test a general decline in plasma n-3 fatty acids and a general increase in plasma n-6 fatty acids were found. These changes were strongest in the chia group (high in n-3), which also resulted in a significant increase in the plasma n-6:n-3 ratio. Similar effects were also found in monkeys, where chronically elevated cortisol concentrations resulted in a decrease of plasma n-3 and MUFAs [32]. Several hormones, but especially glucocorticoids are known to modulate fatty acid metabolism processes [75], [76]. But also the relations between glucocorticoids and food intake and therefore the uptake of specific nutrients and their availability in the body have been discussed [30], [77]. The elevated cortisol concentrations found here may have modified the daily uptake of fatty acids from the diet and therefore affected the fatty acid metabolism. The latter may be finally indicated by the decrease in plasma n-3 and the increase in plasma n-6 fatty acids. A further explanation for the observed changes in the plasma fatty acid status could be energy mobilization via gluconeogenesis from fat tissues for instance, caused by cortisol [29], [77]. This would suggest a vulnerability of n-3 fatty acids to the physiological actions of cortisol following behavioral stressors, as applied here. In the worst case, such a decrease in plasma n-3 fatty acids can impair membrane fatty acid compositions and several metabolic processes in different tissues [78].

Finally, no sex-specific effects related to the supplemented high-UFA diets were detected in this study, neither in behavioral expressions nor in saliva cortisol concentrations. This indicates that males and females were affected in the same way by the dietary high-UFA supplements. However, males generally showed more agonistic and sexual behaviors throughout the social confrontation test, especially on the first day. This was corroborated by elevated cortisol concentrations compared to females, indicating that males are the more proactive sex in guinea pigs. These findings are in line with previous findings and reflect the polygynous mating system with males competing for female monopolizations, accompanied by elevated physiological stress reactions [41], [42]. The strong decline in displayed behaviors from the first to the second day of the social confrontation test might therefore indicate an establishment of a stable social system.

This study does not fully support the importance of diets high in n-3 fatty acids and a lower n-6:n-3 ratio of approximately 4∶1 alone, as found in the plasma of the chia group. Our results rather argue for positive effects of PUFAs in general, including n-3 and n-6 fatty acids, as positive effects on physiology and behavior were found for the chia (high in the n-3 ALA) and walnut groups (high in the n-6 LA), showing quite different n-6:n-3 ratios but also elevated P:S ratios in plasma. However, n-9 MUFAs seem to have a low contribution regarding the modulation of neurobiological mechanisms in guinea pigs, as no effects were found on behavioral and physiological stress responses at all. Therefore, we finally conclude that dietary supplements high in PUFAs can play a positive role in modulating behavioral and physiological stress responses in a social environment, and glucocorticoids may affect the plasma n-3 and n-6 fatty acid status, indicating an interaction of UFAs and physiological stress in guinea pigs.

## Supporting Information

S1 Table Nutrients of total provided and supplemented foods per group. Calculations based on the manufacturer’s information for the Altromin 3023 guinea pig pellets and the US Department of Agriculture National Nutrient Database. Grubb’s test was performed to control for deviations between groups and additionally the standard deviation (SD) in percentage of the mean is stated to illustrate the actual deviation for each nutrient among the experimental diets.
(DOCX)

S2 Table Statistics for removed terms/interactions in order of their removal during model fitting (based on the AIC) at the last appearance in the model.
(DOCX)

S1 Dataset **Behavioral and physiological data for single individuals.**
(PDF)
