# Correction: Effects of Diets High in Unsaturated Fatty Acids on Socially Induced Stress Responses in Guinea Pigs

**DOI:** 10.1371/journal.pone.0120188

**Published:** 2015-03-20

**Authors:** The PLOS ONE Staff

The third author’s name is abbreviated incorrectly in the citation. The correct citation is: Nemeth M, Millesi E, Wagner KH, Wallner B (2014) Effects of Diets High in Unsaturated Fatty Acids on Socially Induced Stress Responses in Guinea Pigs. PLoS ONE 9(12): e116292. doi:10.1371/journal.pone.0116292

There is an error in affiliation 2 for Karl-Heinz Wagner. Affiliation 2 should be: Department of Nutritional Sciences, University of Vienna, Vienna, Austria
