# Supplementary material for: Effects of Diets High in Unsaturated Fatty Acids on Socially Induced Stress Responses in Guinea Pigs
Source: PLoS One. 2014 Dec 31;9(12):e116292. doi: 10.1371/journal.pone.0116292 (PMC4281161; doi:10.1371/journal.pone.0116292)
Supplement: S1 Table — Nutrients of total provided and supplemented foods per group. Calculations based on the manufacturer’s information for the Altromin 3023 guinea pig pellets and the US Department of Agriculture National Nutrient Database. Grubb’s test was performed to control for deviations between groups and additionally the standard deviation (SD) in percentage of the mean is stated to illustrate the actual deviation for each nutrient among the experimental diets. (DOCX) [file pone.0116292.s001.docx]

**Table S1: Nutrients of total provided and supplemented foods per group.** Calculations based on the manufacturer’s information for the Altromin 3023 guinea pig pellets and the US Department of Agriculture National Nutrient Database. Grubb’s test was performed to control for deviations between groups and additionally the standard deviation (SD) in percentage of the mean is stated to illustrate the actual deviation for each nutrient among the experimental diets.

| **Nutrient** | **Unit** | **Group** | | | | **Grubbs-Test** | | **% SD** |
| --- | --- | --- | --- | --- | --- | --- | --- | --- |
|  |  | **Chia** | **Walnut** | **Peanut** | **Control** | **U** | **p** |  |
| **Proximates** |  |  |  |  |  |  |  |  |
| Energy | kcal | 76.105 | 76.945 | 76.51 | 73.675 | 0.055 | 0.056 | 1.930 |
| Total protein | g | 4.9202 | 4.91365 | 4.9665 | 4.8375 | 0.194 | 0.204 | 1.088 |
| Total lipid | g | 1.27245 | 1.4448 | 1.36495 | 1.11875 | 0.253 | 0.272 | 10.767 |
| Ash | g | 2.59025 | 2.57515 | 2.5779 | 2.56625 | 0.252 | 0.27 | 0.385 |
| Total carbohydrate | g | 8.30185 | 8.1598 | 8.1719 | 8.09125 | 0.163 | 0.17 | 1.075 |
| Fiber | g | 4.2295 | 4.091 | 4.1 | 4.0575 | 0.059 | 0.059 | 1.835 |
| Total sugars | g | 0.80125 | 0.8143 | 0.8211 | 0.80125 | 0.387 | 0.434 | 1.222 |
| Starch | g | 0.2827 | 0.2831 | 0.2836 | 0.2825 | 0.209 | 0.218 | 0.172 |
| **Minerals** |  |  |  |  |  |  |  |  |
| Calcium, Ca | mg | 253.96125 | 252.29625 | 252.26625 | 251.80625 | 0.149 | 0.155 | 0.568 |
| Iron, Fe | mg | 5.3471 | 5.32305 | 5.3314 | 5.3085 | 0.344 | 0.381 | 0.303 |
| Magnesium, Mg | mg | 73.7975 | 72.9125 | 72.9625 | 72.1225 | 0.316 | 0.346 | 0.938 |
| Phosphorus, P | mg | 258.725 | 256.155 | 256.305 | 254.425 | 0.233 | 0.248 | 0.689 |
| Potassium, K | mg | 367.116 | 367.286 | 368.606 | 365.081 | 0.209 | 0.222 | 0.397 |
| Sodium, Na | mg | 74.67475 | 74.60475 | 74.68475 | 74.59475 | 0.585 | 0.711 | 0.062 |
| Zinc, Zn | mg | 1.64615 | 1.6387 | 1.6396 | 1.62325 | 0.117 | 0.121 | 0.593 |
| Copper, Cu | mg | 0.37887 | 0.38218 | 0.37997 | 0.37425 | 0.17 | 0.178 | 0.882 |
| Manganese, Mn | mg | 1.852615 | 1.85607 | 1.84867 | 1.839 | 0.168 | 0.176 | 0.399 |
| Sulfur, S | mg | 51.71 | 51.71 | 51.71 | 51.71 | - | - | 0 |
| Chlorine, Cl | mg | 115.592 | 115.592 | 115.592 | 115.592 | - | - | 0 |
| Iodine, I | mg | 0.0328 | 0.0328 | 0.0328 | 0.0328 | - | - | 0 |
| Molybdenum, Mo | mg | 0.02435 | 0.02435 | 0.02435 | 0.02435 | - | - | 0 |
| Cobalt | mg | 0.008875 | 0.008875 | 0.008875 | 0.008875 | - | - | 0 |
| Selenium, Se | µg | 12.401 | 12.2495 | 12.161 | 12.125 | 0.082 | 0.084 | 1.055 |
| Fluoride, F | µg | 138.3875 | 138.3875 | 138.3875 | 138.3875 | - | - | 0 |
| **Vitamins** |  |  |  |  |  |  |  |  |
| Vitamin C | mg | 40.9655 | 40.964 | 40.9575 | 40.9575 | 0.525 | 0.621 | 0.010 |
| Thiamin | mg | 0.014725 | 0.01333 | 0.014825 | 0.011625 | 0.207 | 0.219 | 10.998 |
| Riboflavin | mg | 0.012225 | 0.012125 | 0.01205 | 0.011375 | 0.095 | 0.097 | 3.231 |
| Niacin | mg | 0.179275 | 0.14075 | 0.195455 | 0.135125 | 0.446 | 0.511 | 18.064 |
| Pantothenic acid | mg | 0.9415 | 0.94435 | 0.947335 | 0.9415 | 0.232 | 0.248 | 0.295 |
| Vitamin B-6 | mg | 0.39725 | 0.399935 | 0.39899 | 0.39725 | 0.378 | 0.423 | 0.335 |
| Folate | µg | 3.495 | 3.74 | 4.45 | 3.25 | 0.149 | 0.155 | 13.866 |
| Folic acid | µg | 118.95 | 118.95 | 118.95 | 118.95 | - | - | 0 |
| Choline | mg | 1.85 | 2.046 | 2.1125 | 1.85 | 0.468 | 0.541 | 6.877 |
| Betaine | mg | 0.0625 | 0.064 | 0.0655 | 0.0625 | 0.242 | 0.259 | 2.257 |
| Vitamin B-12 | µg | 1 | 1 | 1 | 1 | - | - | 0 |
| Carotene | µg | 1477.25 | 1477.25 | 1477.25 | 1477.25 | - | - | 0 |
| Cryptoxanthin | µg | 3.25 | 3.25 | 3.25 | 3.25 | - | - | 0 |
| Vitamin A | IU | 2726.645 | 2726.475 | 2726.375 | 2726.375 | 0.137 | 0.142 | 0.005 |
| Lycopene | µg | 0.125 | 0.125 | 0.125 | 0.125 | - | - | 0 |
| Lutein | µg | 34.875 | 34.875 | 34.875 | 34.875 | - | - | 0 |
| Vitamin E | mg | 3.23125 | 3.25225 | 3.2704 | 3.22875 | 0.099 | 0.101 | 0.613 |
| Vitamin D | IU | 25 | 25 | 25 | 25 | - | - | 0 |
| Vitamin K | µg | 503.7 | 503.7 | 503.7 | 503.7 | - | - | 0 |
| **Lipids** |  |  |  |  |  |  |  |  |
| 14:0 | g | 0.000775 | 0.000625 | 0.00075 | 0.000625 | 0.542 | 0.647 | 11.537 |
| 16:0 | g | 0.086225 | 0.097395 | 0.101145 | 0.075375 | 0.296 | 0.322 | 12.937 |
| 18:0 | g | 0.025685 | 0.02942 | 0.026625 | 0.021125 | 0.212 | 0.225 | 13.401 |
| 20:0 | g | 0.002465 | 0.002315 | 0.002 | 0.002 | 0.405 | 0.457 | 10.631 |
| Total SFA | g | 0.11565 | 0.12963 | 0.14217 | 0.099 | 0.341 | 0.376 | 15.264 |
| 16:1 | g | 0.002645 | 0.0025 | 0.002545 | 0.0025 | 0.096 | 0.099 | 2.684 |
| 18:1 n-9 | g | 0.13039 | 0.16337 | 0.238155 | 0.119375 | 0.033 | 0.033 | 32.912 |
| 20:1 n-9 | g | 0.00273 | 0.00317 | 0.005805 | 0.0025 | 0.122 | 0.126 | 43.027 |
| Total MUFA | g | 0.13367 | 0.16679 | 0.24427 | 0.122125 | 0.118 | 0.122 | 33.027 |
| 18:2 n-6 | g | 0.3243 | 0.48559 | 0.3729 | 0.295125 | 0.147 | 0.152 | 22.678 |
| 18:3 n-3 | g | 0.128275 | 0.084525 | 0.03914 | 0.039125 | 0 | 0.009 | 58.746 |
| Total PUFA | g | 0.45245 | 0.569995 | 0.41192 | 0.334125 | 0.249 | 0.267 | 22.251 |
| Total UFA | g | 0.58612 | 0.736785 | 0.65619 | 0.45625 | 0.485 | 0.565 | 19.529 |
| M:S | ratio | 1.15581496 | 1.28666204 | 1.71815432 | 1.23358586 | 0.045 | 0.046 | 18.701 |
| P:S | ratio | 3.91223519 | 4.39709172 | 2.89737638 | 3.375 | 0.406 | 0.459 | 17.840 |
| U:S | ratio | 5.06805015 | 5.68375376 | 4.6155307 | 4.60858586 | 0 | 0.012 | 10.165 |
| n-6:n-3 | ratio | 2.52816215 | 5.74492754 | 9.52733776 | 7.54313099 | 0.27 | 0.292 | 46.901 |
| **Amino acids** |  |  |  |  |  |  |  |  |
| Tryptophan | g | 0.067305 | 0.065975 | 0.066375 | 0.065125 | 0.332 | 0.365 | 1.367 |
| Threonine | g | 0.197795 | 0.19723 | 0.198665 | 0.19425 | 0.095 | 0.097 | 0.973 |
| Isoleucine | g | 0.200005 | 0.199125 | 0.200535 | 0.196 | 0.082 | 0.084 | 1.020 |
| Leucine | g | 0.33773 | 0.336725 | 0.339235 | 0.330875 | 0.08 | 0.081 | 1.089 |
| Lysine | g | 0.2231 | 0.22037 | 0.22288 | 0.21825 | 0.291 | 0.316 | 1.038 |
| Methionine | g | 0.09044 | 0.08868 | 0.089085 | 0.0875 | 0.307 | 0.336 | 1.364 |
| Cystine | g | 0.06916 | 0.068165 | 0.06878 | 0.067125 | 0.213 | 0.226 | 1.301 |
| Phenylalanine | g | 0.21183 | 0.210305 | 0.213435 | 0.20675 | 0.2 | 0.212 | 1.356 |
| Tyrosine | g | 0.152315 | 0.15153 | 0.154745 | 0.1495 | 0.299 | 0.326 | 1.426 |
| Valine | g | 0.228875 | 0.22789 | 0.229535 | 0.224125 | 0.078 | 0.08 | 1.062 |
| Arginine | g | 0.31084 | 0.311515 | 0.31555 | 0.300125 | 0.1 | 0.102 | 2.130 |
| Histidine | g | 0.111155 | 0.110455 | 0.11176 | 0.1085 | 0.142 | 0.147 | 1.282 |
| Alanine | g | 0.232595 | 0.230855 | 0.2325 | 0.227375 | 0.107 | 0.11 | 1.056 |
| Aspartic acid | g | 0.47057 | 0.47127 | 0.477855 | 0.462125 | 0.259 | 0.278 | 1.371 |
| Glutamic acid | g | 0.816 | 0.81258 | 0.82545 | 0.7985 | 0.237 | 0.254 | 1.374 |
| Glycine | g | 0.21309 | 0.212455 | 0.216145 | 0.208375 | 0.254 | 0.273 | 1.504 |
| Proline | g | 0.246755 | 0.246405 | 0.248565 | 0.242875 | 0.158 | 0.165 | 0.967 |
| Serine | g | 0.224495 | 0.22392 | 0.225605 | 0.21925 | 0.062 | 0.063 | 1.254 |
