# Supplementary material for: Effects of Diets High in Unsaturated Fatty Acids on Socially Induced Stress Responses in Guinea Pigs
Source: PLoS One. 2014 Dec 31;9(12):e116292. doi: 10.1371/journal.pone.0116292 (PMC4281161; doi:10.1371/journal.pone.0116292)
Supplement: S2 Table — Statistics for removed terms/interactions in order of their removal during model fitting (based on the AIC) at the last appearance in the model. (DOCX) [file pone.0116292.s002.docx]

**Table S2: Statistics for removed terms/interactions in order of their removal during model fitting (based on the AIC) at the last appearance in the model.**

| **Response variable** | **Predictor** | **Statistics** | | |
| --- | --- | --- | --- | --- |
|  |  | **df** | **F-statistic** | **p-value** |
| Locomotion | Group:Sex:Day | 6,142 | 1.497 | 0.183 |
|  | Group:Day | 6,148 | 0.161 | 0.987 |
|  | Group:Sex | 3,71 | 0.009 | 0.999 |
| Socio-positive behavior | Group:Sex:Day | 6,142 | 0.344 | 0.912 |
|  | Group:Day | 6,148 | 0.509 | 0.801 |
|  | Group:Sex | 3,71 | 0.168 | 0.918 |
|  | Group | 3,74 | 0.460 | 0.711 |
|  | Sex:Day | 2,154 | 0.571 | 0.566 |
|  | Sex | 1,77 | 0.513 | 0.476 |
|  | Day | 2,156 | 1.280 | 0.281 |
| Agonistic behavior | Group:Sex:Day | 6,142 | 1.300 | 0.261 |
|  | Group:Day | 6,148 | 0.637 | 0.701 |
|  | Group:Sex | 3,71 | 0.758 | 0.522 |
|  | Group | 3,74 | 1.435 | 0.239 |
| Sexual behavior | Group:Sex:Day | 61,142 | 1.155 | 0.334 |
|  | Group:Day | 6,148 | 0.383 | 0.889 |
|  | Group:Sex | 3,71 | 0.566 | 0.639 |
|  | Group | 3,74 | 0.533 | 0.661 |
| Saliva cortisol concentrations | Group:Sex:Day | 12,284 | 0.679 | 0.771 |
|  | Group:Day | 12,296 | 1.176 | 0.300 |
|  | Group:Sex | 3,71 | 0.526 | 0.666 |
|  | Sex:Day | 4,308 | 1.396 | 0.235 |
| Bodyweight | Group:Sex:Day | 12,284 | 0.641 | 0.807 |
|  | Group:Sex | 3,71 | 0.001 | 1.000 |
|  | Sex:Day | 4,296 | 0.824 | 0.511 |
|  | Group:Day | 12,300 | 1.857 | 0.039 |
|  | Group | 3,74 | 0.866 | 0.463 |
|  | Sex | 1,77 | 1.214 | 0.274 |
