# Supplementary material for: Effects of Diets High in Unsaturated Fatty Acids on Socially Induced Stress Responses in Guinea Pigs
Source: PLoS One. 2014 Dec 31;9(12):e116292. doi: 10.1371/journal.pone.0116292 (PMC4281161; doi:10.1371/journal.pone.0116292)
Supplement: S1 Dataset — Behavioral and physiological data for single individuals. (PDF) [file pone.0116292.s003.pdf]

Dataset S1: Behavioral and physiological data for single individuals.

Code	Group	Sex	Move_Soc1	Move_Soc2	Move_Soc3	Soc_Soc1	Soc_Soc2	Soc_Soc3
Sm1	SeedsChia	m	761.28	466.64	109.4	2	11	7
Sm2	SeedsChia	m	49.72	59.28	29.16	3	17	15
Sm3	SeedsChia	m	557.04	331.24	155.64	7	13	13
Sm4	SeedsChia	m	309.16	221.12	169.72	6	33	36
Sm5	SeedsChia	m	522.48	146.6	100	6	13	11
Sm6	SeedsChia	m	261.12	5.24	28.24	15	1	10
Sm7	SeedsChia	m	316.36	301.48	51.88	57	51	12
Sm8	SeedsChia	m	457.44	40.72	5.12	36	4	9
Sm9	SeedsChia	m	891.56	152.6	136.32	25	37	31
Sm10	SeedsChia	m	359.32	23.12	120.8	69	8	45
Sf1	SeedsChia	f	140.72	77.2	33.2	2	7	2
Sf2	SeedsChia	f	77.8	105.88	90.6	4	12	6
Sf3	SeedsChia	f	84.36	174.48	52.76	4	18	23
Sf4	SeedsChia	f	30.64	71.44	41.56	2	33	21
Sf5	SeedsChia	f	69.64	67.52	83.12	7	13	17
Sf6	SeedsChia	f	18.92	69.84	93.28	15	12	16
Sf7	SeedsChia	f	380.32	150.44	24.8	30	23	10
Sf8	SeedsChia	f	129.32	28.24	2	31	10	10
Sf9	SeedsChia	f	58.24	40.68	60.6	15	15	15
Sf10	SeedsChia	f	163	28.48	134.28	22	21	28
Wm1	Walnut	m	206.44	175.24	68.32	12	1	2
Wm2	Walnut	m	675.8	373.76	185.84	2	14	13
Wm3	Walnut	m	139.88	50.92	74.84	3	2	2
Wm4	Walnut	m	372.6	154.76	97.24	5	20	2
Wm5	Walnut	m	750.48	182.16	138.6	10	4	14
Wm6	Walnut	m	667.32	160.2	136.68	22	12	19
Wm7	Walnut	m	387.56	88.28	96.4	50	33	8
Wm8	Walnut	m	783.4	160.44	28	42	26	14
Wm9	Walnut	m	314.84	78.52	75.64	29	9	25
Wm10	Walnut	m	366.32	42.16	113.16	45	17	28
Wf1	Walnut	f	47.08	224.64	16.84	8	6	3
Wf2	Walnut	f	152.24	198.24	75.68	3	4	5
Wf3	Walnut	f	95.8	152.88	42.92	4	19	7
Wf4	Walnut	f	38.72	98.6	66.44	4	36	7
Wf5	Walnut	f	20.2	24.92	24.68	5	17	7
Wf6	Walnut	f	63.72	30.56	27.16	10	19	6
Wf7	Walnut	f	296.4	68.32	67.08	22	15	17
Wf8	Walnut	f	97.88	70.28	872.72	15	8	4
Wf9	Walnut	f	117.68	65.2	43.88	17	26	21
Wf10	Walnut	f	211.6	29.44	159.28	65	6	52
Pm1	Peanut	m	103.4	49.16	13.64	2	4	0
Pm2	Peanut	m	227.88	116.44	105.28	4	7	8
Pm3	Peanut	m	174.44	90.36	59.64	10	11	2
Pm4	Peanut	m	99.2	146.12	87.88	19	36	26
Pm5	Peanut	m	398.96	63.16	19.04	12	16	0
Pm6	Peanut	m	98.96	11.04	65.4	30	12	22
Pm7	Peanut	m	679.4	147.76	318.64	28	22	29
Pm8	Peanut	m	133.32	101	52.84	6	23	8
Pm9	Peanut	m	229.84	81.08	41.32	29	5	9
Pm10	Peanut	m	406.56	124.88	498.88	36	14	87
Pf1	Peanut	f	36.8	58.32	2.28	3	5	6
Pf2	Peanut	f	24.93	61.04	13	4	9	3
Pf3	Peanut	f	65.04	10.92	43.8	6	11	10
Pf4	Peanut	f	45.68	28.88	33.36	1	23	10
Pf5	Peanut	f	10.56	28.2	6.44	6	8	10
Pf6	Peanut	f	142.96	40.6	29.32	18	12	8
Pf7	Peanut	f	186.8	52.4	77.68	30	26	19
Pf8	Peanut	f	78.08	75.04	10.92	18	20	6
Pf9	Peanut	f	233.44	57.6	38.8	49	27	19
Pf10	Peanut	f	302.2	31.68	60.28	89	3	31
Cm1	Control	m	16.64	16.4	71.24	4	5	3
Cm2	Control	m	149.92	299.28	128.56	3	13	10
Cm3	Control	m	377.04	209.2	91.16	1	9	8
Cm4	Control	m	642.28	102.48	140.6	14	20	14
Cm5	Control	m	365.08	214.88	157.24	12	29	34
Cm6	Control	m	30.76	9.24	33.32	8	6	7
Cm7	Control	m	329.48	176.76	38.44	48	42	7
Cm8	Control	m	289.76	40.92	2.44	23	10	4
Cm9	Control	m	169.16	54.48	48.08	22	21	12
Cm10	Control	m	550.8	58.04	104.28	43	5	26
Cf1	Control	f	42.44	187.68	8.44	4	9	5
Cf2	Control	f	29.2	16.6	0	3	3	2
Cf3	Control	f	26	1.48	24.92	3	6	11
Cf4	Control	f	172.88	84.2	98.28	2	15	17
Cf6	Control	f	39.96	43.96	37	9	7	10
Cf7	Control	f	196.6	164.36	29.8	32	39	7
Cf8	Control	f	67.4	117.28	29.36	25	21	8
Cf9	Control	f	92	5.08	47	13	4	14
Cf10	Control	f	251.8	32.84	69.84	45	5	13

Aggr_Soc1	Aggr_Soc2	Aggr_Soc3	Sex_Soc1	Sex_Soc2	Sex_Soc3	Cort_pre	Cort_Soc1
71	11	3	117	70	15	48.45	64.06
7	1	1	0	3	2	59.57	41.40
101	16	18	151	47	16	37.31	116.14
17	19	7	3	26	30	16.76	67.50
27	0	0	4	4	1	19.04	66.07
34	0	1	61	0	6	57.64	134.73
22	66	2	83	72	3	50.15	130.25
31	1	0	93	2	0	58.30	45.93
219	22	24	225	34	32	1.59	65.29
31	1	2	77	0	18	21.73	31.07
5	3	6	0	0	0	9.51	84.78
1	2	2	0	2	2	12.02	40.14
7	0	0	1	4	2	32.28	41.11
6	4	5	0	4	1	58.48	101.98
5	2	3	0	0	0	52.33	60.80
1	3	2	0	3	5	41.31	27.81
10	12	1	6	13	0	27.69	51.52
4	7	1	1	1	1	56.64	70.07
2	7	17	0	1	1	7.49	11.81
6	2	1	1	2	4	3.30	15.83
20	5	3	39	17	7	35.33	78.78
92	39	2	89	45	5	51.08	69.75
14	3	12	5	1	6	51.97	57.41
82	1	1	56	21	4	90.63	185.35
69	11	6	108	6	9	10.76	49.91
87	5	13	178	43	27	84.16	135.63
26	8	24	80	14	14	92.29	163.78
211	20	3	149	31	0	81.86	47.15
33	14	2	36	8	8	3.20	56.24
22	3	2	50	13	5	21.13	32.18
2	4	1	0	2	1	107.51	39.78
5	6	1	0	1	0	0.78	32.63
10	8	2	0	5	2	15.66	54.41
11	2	0	0	6	2	37.12	95.26
4	2	0	0	1	0	24.21	49.52
1	4	0	0	1	2	50.72	100.89
15	13	5	7	4	5	5.24	26.25
11	9	0	2	0	0	81.27	54.20
1	13	8	0	4	7	6.82	45.17
18	6	47	28	0	4	16.93	25.16
4	4	0	3	2	0	60.76	108.67
22	1	21	11	2	18	63.18	101.50
10	4	1	19	17	1	84.88	85.55
29	9	5	6	26	19	73.17	103.03
56	3	0	12	4	0	42.94	40.44
15	1	6	17	1	4	58.02	104.23
94	4	33	209	11	77	125.73	226.53
15	3	2	9	15	4	45.27	27.97
19	1	2	56	2	3	3.62	32.65
41	3	54	60	19	96	26.18	61.78
6	3	2	0	0	0	17.69	84.46
4	7	3	0	3	0	43.68	60.14
7	0	1	1	0	2	33.97	41.43
5	8	11	0	0	0	45.51	163.42
1	5	3	0	0	0	34.49	53.65
10	1	2	0	6	2	33.38	128.61
11	9	5	5	1	6	8.35	19.33
3	15	0	4	6	1	28.27	42.43
7	16	5	7	8	2	15.50	27.64
27	6	9	29	1	4	47.10	67.57
0	0	0	0	0	0	35.33	235.33
17	27	38	34	44	41	82.55	56.60
30	5	2	51	27	13	65.42	104.44
134	3	4	84	13	26	77.92	214.75
47	30	22	53	47	32	395.29	156.31
3	0	1	1	3	0	9.66	95.16
16	17	2	108	39	0	28.64	269.87
8	5	1	33	0	0	25.35	109.20
10	9	0	9	5	4	34.41	48.45
70	0	13	107	4	20	12.34	25.16
4	4	0	0	8	1	34.66	127.40
10	0	0	0	0	0	20.07	84.81
3	0	1	0	1	0	70.94	100.31
16	17	8	1	5	4	85.49	87.76
7	3	0	0	3	5	31.08	47.88
4	1	1	3	5	0	150.20	56.65
1	30	2	1	6	7	44.94	58.57
4	0	8	0	0	0	7.29	55.75
29	1	5	10	3	3	1.16	43.64

Cort_Soc2	Cort_Soc3	Cort_post	BW_pre	BW_Soc1	BW_Soc2	BW_Soc3	BW_post
267.50	36.35	16.23	750	740	739	782	793
46.44	42.42	31.18	801	792	797	807	813
201.45	63.04	53.60	644	655	639	648	661
17.43	93.33	5.97	809	817	766	754	789
49.13	44.47	16.67	714	713	693	700	716
114.98	79.91	28.02	839	839	797	798	810
99.84	118.57	35.40	704	694	664	661	704
58.57	93.26	18.24	630	633	599	613	643
0.72	33.01	6.88	803	779	735	746	740
39.85	48.63	17.30	582	577	550	558	585
82.31	72.55	18.79	746	747	728	745	754
21.71	80.22	60.88	917	914	870	875	878
66.26	36.82	25.91	846	833	800	802	823
52.74	45.00	44.89	860	860	839	843	849
83.74	110.41	26.70	1009	1012	1008	999	1008
47.36	66.91	27.29	613	608	585	610	609
91.90	43.36	266.78	666	671	648	632	620
83.65	97.22	8.54	545	545	534	520	567
30.98	51.97	19.01	712	712	674	690	701
10.30	21.36	4.47	672	653	642	667	692
92.03	60.57	57.81	707	710	673	693	713
84.81	67.54	350.03	854	834	809	827	845
238.84	148.13	56.08	761	764	720	736	767
91.75	56.43	33.70	639	634	613	629	641
50.72	35.76	17.96	919	923	885	878	894
81.07	66.44	45.23	808	804	778	795	803
76.77	40.12	105.62	763	770	758	773	807
68.89	56.59	21.88	785	780	756	767	805
42.85	36.92	9.25	604	609	554	563	597
19.68	45.00	17.92	534	525	518	545	545
45.64	42.81	7.45	819	821	792	805	802
77.57	122.50	18.68	870	868	823	828	863
123.36	217.97	5.14	812	818	793	794	795
171.97	59.40	7.20	773	778	758	772	791
211.96	81.62	29.14	1162	1156	1151	1127	1165
79.76	58.62	43.15	735	742	705	710	716
19.70	19.09	58.30	633	649	611	619	658
45.53	37.45	17.01	727	735	710	710	750
19.64	32.41	14.80	578	569	552	553	571
12.21	41.39	21.19	578	573	566	563	574
132.91	128.48	62.68	702	693	656	663	652
188.12	78.62	34.88	769	751	705	720	744
194.75	195.69	55.47	659	671	638	664	691
84.80	5.00	24.58	903	899	820	812	861
31.74	66.53	51.28	661	657	675	663	668
88.45	94.49	81.65	764	768	739	745	777
101.24	64.98	47.46	788	771	745	743	790
34.22	171.73	5.52	740	741	713	705	723
184.18	180.96	17.31	647	645	583	588	633
65.16	81.36	27.96	572	563	538	535	565
70.98	77.72	26.18	749	753	705	728	750
110.35	314.70	137.13	1073	1067	1029	1040	1049
210.52	102.02	24.49	887	893	860	865	870
176.72	124.11	26.67	701	707	683	672	698
92.86	210.78	32.29	925	915	903	900	920
50.76	95.89	10.57	648	652	611	626	632
19.83	20.32	2.19	630	657	624	613	654
39.51	72.49	1.42	590	592	559	569	607
108.67	261.95	14.58	564	570	542	541	571
28.59	34.90	21.19	644	633	613	625	656
463.86	260.94	78.50	631	623	610	610	612
144.72	66.73	105.62	884	896	867	871	895
40.84	127.60	42.38	606	623	607	635	636
83.36	103.03	123.65	630	635	621	630	649
59.82	216.75	65.72	949	952	920	913	929
321.10	61.11	20.29	806	808	774	804	803
31.42	18.27	2.51	620	626	593	592	638
109.20	109.20	16.28	516	515	508	507	548
48.45	62.49	21.28	544	541	524	535	560
44.32	29.90	6.28	543	541	511	511	550
170.16	88.24	5.40	810	805	777	787	780
40.74	33.47	75.54	739	738	702	706	727
229.24	249.00	85.65	621	623	589	617	648
139.81	112.89	90.62	884	898	883	885	911
114.87	80.49	21.10	721	730	699	711	739
41.83	59.90	3.00	582	615	593	607	641
67.14	75.70	1.63	640	640	612	620	645
2.91	9.41	12.86	739	733	699	722	743
35.60	27.55	14.18	580	573	572	561	576

C16:0_pre	C16:0_post	C18:0_pre	C18:0_post	C18:1n-9_pre	C18:1n-9_post	C20:1n-9_pre
15.91	12.42	12.57	13.15	13.49	12.26	0.22
13.13	14.44	9.83	12.31	10.49	9.66	0.18
11.89	12.85	11.00	11.18	11.71	13.28	0.00
15.29	16.46	13.41	12.18	14.65	16.05	0.23
15.23	15.51	12.08	12.89	13.40	14.20	0.19
0.00	12.09	0.00	13.15	0.00	10.94	0.00
16.80	0.00	13.97	0.00	13.99	0.00	0.02
16.30	15.11	12.01	15.85	12.16	11.49	0.05
13.82	14.31	11.31	13.83	10.38	12.15	0.08
13.56	12.88	12.15	11.61	11.57	10.06	0.04
15.60	13.59	10.41	11.78	13.76	12.15	0.11
13.55	13.46	10.66	10.77	12.87	13.10	0.20
15.07	15.85	10.56	8.74	13.75	13.95	0.13
14.77	14.01	9.31	10.28	11.83	11.86	0.12
15.32	15.59	10.65	10.80	15.50	13.70	0.12
14.83	14.47	13.66	10.38	11.18	12.86	0.23
17.48	19.14	11.33	11.27	14.16	15.76	0.02
13.46	13.40	13.26	13.74	10.72	10.52	0.10
15.14	13.77	12.33	11.93	1.23	11.86	0.12
12.80	11.39	14.11	13.62	12.56	11.94	0.02
12.80	11.45	13.82	12.00	10.85	11.97	0.08
9.88	11.85	9.02	14.78	13.85	9.24	0.18
0.00	11.61	0.00	11.65	0.00	13.04	0.00
16.12	15.38	8.04	9.54	15.03	13.75	0.15
13.19	14.91	12.07	10.75	12.49	13.63	0.16
0.00	10.95	0.00	12.75	0.00	11.04	0.00
17.00	0.00	12.55	0.00	12.35	0.00	0.10
13.02	12.88	11.76	11.21	10.41	9.15	0.04
15.98	13.01	12.07	13.52	11.89	10.35	0.44
1.79	12.38	13.62	10.93	12.22	12.02	0.02
15.31	17.00	8.28	7.78	16.57	18.81	0.14
13.24	14.19	8.98	9.96	11.22	11.21	0.11
11.85	11.76	11.44	11.15	11.64	12.97	0.11
11.77	13.48	9.53	8.63	11.82	11.66	0.23
14.41	14.14	7.27	7.89	19.83	21.29	0.14
14.84	13.79	11.80	11.31	11.66	10.68	0.01
17.52	14.11	13.85	12.11	12.26	12.51	0.00
13.79	13.05	11.74	11.65	11.23	11.19	0.09
12.31	12.28	9.99	10.66	11.35	10.97	0.20
13.08	12.91	12.35	10.15	11.31	11.86	0.10
13.39	11.81	10.45	11.15	17.09	15.23	0.19
11.01	11.46	10.51	11.23	19.35	12.88	0.29
11.16	11.84	10.47	11.85	20.24	17.29	0.40
13.08	17.26	11.96	11.95	17.05	21.91	0.17
12.98	11.96	12.78	13.52	15.73	15.76	0.15
14.92	13.02	13.12	11.22	17.80	24.73	0.29
18.26	14.09	12.34	10.97	13.14	22.00	0.05
12.67	12.97	11.97	13.37	22.07	12.62	0.35
12.24	13.37	10.36	10.55	20.70	18.16	0.25
13.02	13.31	11.69	12.05	20.96	14.21	0.20
13.48	13.64	10.22	10.24	18.49	16.24	0.26
13.98	14.41	11.42	10.10	13.28	13.21	0.19
13.00	13.27	11.80	10.53	16.57	15.04	0.25
12.37	12.80	11.43	12.16	12.78	13.86	0.18
15.44	15.56	7.83	9.81	17.09	15.06	0.25
0.00	13.75	0.00	10.48	0.00	17.57	0.00
16.61	14.74	12.06	10.44	12.72	20.36	0.10
13.03	14.07	12.44	11.21	16.34	17.89	0.14
13.15	11.37	10.63	10.23	20.30	23.30	0.46
13.08	13.20	12.93	12.05	14.37	19.02	0.15
15.22	15.27	9.81	12.48	12.53	12.56	0.29
14.44	15.05	9.36	10.75	14.16	12.17	0.00
4.86	6.16	22.26	19.56	14.44	14.99	0.53
8.63	7.31	14.19	17.34	14.69	13.25	0.24
16.44	16.53	11.94	11.90	12.58	12.85	0.19
17.66	13.79	13.27	13.42	9.86	11.65	0.52
18.18	14.94	14.92	13.25	11.67	11.39	0.13
15.34	14.56	11.11	12.77	11.75	10.57	0.08
13.90	14.87	10.93	13.86	12.53	11.34	0.17
14.09	13.60	13.31	12.87	11.10	10.88	0.06
14.95	14.46	9.46	8.64	12.60	12.49	0.17
15.44	15.05	10.68	9.94	12.32	12.95	0.18
14.76	14.32	8.69	10.33	10.82	11.52	0.14
15.48	15.29	10.75	11.24	13.75	13.89	0.15
17.67	16.12	13.01	12.24	13.94	14.34	0.10
18.73	16.13	12.15	12.84	12.79	14.58	0.03
14.29	15.13	12.60	12.71	11.74	12.13	0.09
15.98	15.72	12.06	10.58	11.89	12.18	0.44
15.60	13.78	12.14	12.03	12.01	11.64	0.11

C20:1n-9_post	C18:2n-6_pre	C18:2n-6_post	C20:2n-6_pre	C20:2n-6_post	C20:4n-6_pre
0.22	45.80	46.16	0.35	0.28	2.41
0.14	49.23	50.43	0.18	0.15	2.90
0.00	46.42	50.29	0.28	0.34	1.69
0.13	39.06	38.74	0.28	0.18	2.05
0.17	41.55	42.62	0.24	0.16	2.15
0.28	0.00	44.55	0.00	0.53	0.00
0.00	39.09	0.00	0.36	0.00	2.28
0.12	35.77	39.44	0.34	0.33	2.22
0.28	39.91	43.43	0.32	0.31	1.86
0.11	40.08	42.42	0.34	0.29	2.38
0.20	46.92	48.87	0.22	0.36	2.55
0.19	49.53	53.57	0.34	0.27	1.89
0.21	46.49	42.91	0.26	0.19	2.13
0.11	48.72	51.28	0.22	0.24	1.99
0.00	45.57	46.09	0.25	0.24	3.08
0.11	40.25	42.19	0.40	0.33	2.83
0.04	38.10	36.46	0.25	0.21	1.88
0.10	39.25	42.82	0.51	0.38	2.78
0.14	49.56	43.77	0.35	0.39	2.35
0.06	38.45	43.55	0.22	0.35	3.31
0.15	48.77	51.28	0.19	0.27	3.13
0.05	55.16	52.05	0.21	0.25	1.67
0.17	0.00	50.98	0.00	0.28	0.00
0.11	48.26	49.46	0.20	0.17	2.54
0.09	48.32	46.08	0.26	0.20	2.38
0.24	0.00	47.05	0.00	0.10	0.00
0.00	40.51	0.00	0.32	0.00	3.11
0.14	44.48	44.10	0.25	0.21	2.36
0.40	42.28	47.91	0.34	0.38	1.81
0.19	52.69	47.91	0.47	0.44	2.44
0.13	47.29	44.60	0.26	0.24	1.47
0.07	56.14	55.37	0.26	0.20	2.11
0.14	51.82	51.28	0.25	0.26	1.84
0.14	52.23	53.32	0.26	0.37	2.12
0.03	47.06	47.59	0.32	0.09	2.09
0.07	44.57	47.04	0.30	0.26	2.38
0.10	39.48	44.80	0.29	0.36	2.51
0.06	41.29	47.78	0.26	0.27	2.39
0.13	50.10	49.14	0.23	0.52	2.28
0.10	46.89	47.22	0.42	0.58	2.77
0.22	48.35	51.38	0.31	0.22	2.93
0.18	47.94	52.52	0.20	0.38	1.83
0.20	47.21	47.15	0.25	0.31	1.59
0.20	46.34	36.74	0.18	0.14	2.13
0.26	44.74	44.75	0.22	0.40	3.32
0.31	38.45	38.59	0.39	0.34	3.01
0.27	38.73	40.80	0.31	0.33	2.65
0.05	41.61	46.18	0.26	0.23	2.58
0.20	43.78	44.06	0.30	0.25	2.26
0.10	41.59	45.91	0.35	0.29	2.08
0.28	47.62	48.67	0.24	0.25	2.54
0.19	50.19	50.99	0.38	0.32	3.33
0.21	46.96	49.60	0.47	0.40	3.19
0.18	51.01	49.14	0.29	0.15	2.99
0.21	43.17	42.30	0.21	0.24	3.43
0.15	0.00	44.95	0.00	0.28	0.00
0.23	38.15	40.54	0.26	0.19	3.54
0.18	41.19	43.62	0.32	0.25	3.17
0.31	40.73	43.02	0.34	0.43	1.80
0.25	42.46	40.20	0.38	0.32	3.40
0.21	48.16	47.47	0.40	0.43	3.88
0.21	49.76	49.21	0.28	0.32	2.14
0.00	45.50	47.65	0.68	0.54	2.72
0.28	48.38	48.12	0.35	0.46	2.33
0.13	43.36	42.83	0.27	0.21	3.12
0.06	41.24	46.35	0.25	0.43	3.51
0.08	35.41	44.06	0.33	0.38	2.87
0.10	41.99	45.73	0.37	0.27	1.99
0.14	46.54	45.04	0.32	0.37	1.48
0.08	45.44	48.40	0.35	0.31	2.71
0.12	49.77	51.68	0.28	0.26	1.73
0.13	47.03	48.38	0.32	0.29	2.88
0.15	51.74	51.07	0.36	0.31	4.17
0.18	45.54	44.90	0.21	0.22	2.18
0.10	39.02	42.12	0.26	0.21	3.14
0.14	39.94	40.99	0.35	0.36	2.70
0.06	41.95	43.94	0.29	0.26	2.98
0.12	42.29	45.02	0.34	0.30	1.81
0.13	40.59	46.89	0.37	0.37	2.00

C20:4n-6_post	C18:3n-3_pre	C18:3n-3_post	C20:5n-3_pre	C20:5n-3_post	C22:5n-3_pre
2.50	5.84	10.20	0.00	0.00	0.37
3.87	9.46	3.93	0.00	0.00	0.46
1.56	14.15	7.66	0.00	0.00	0.00
2.08	10.92	9.81	0.14	0.16	0.38
2.63	10.72	7.48	0.06	0.00	0.32
3.35	0.00	7.87	0.00	0.30	0.00
0.00	5.33	0.00	0.07	0.00	0.31
3.76	11.93	6.93	0.07	0.23	0.32
3.57	13.23	6.69	0.14	0.12	0.31
2.66	12.56	12.46	0.05	0.18	0.35
3.66	6.90	6.02	0.00	0.00	0.34
2.42	8.38	3.52	0.00	0.00	0.23
1.95	7.94	11.54	0.00	0.00	0.38
2.13	9.75	7.05	0.00	0.00	0.29
3.33	5.00	5.78	0.11	0.00	0.81
1.82	9.00	11.55	0.24	0.08	0.57
2.76	7.55	6.54	0.14	0.11	0.26
3.33	8.76	7.49	0.10	0.08	0.37
2.63	8.59	8.77	0.08	0.03	0.39
3.02	11.73	9.77	0.10	0.12	0.62
2.94	5.43	5.58	0.00	0.00	0.29
3.76	6.98	3.27	0.00	0.00	0.22
2.26	0.00	5.65	0.00	0.07	0.00
3.26	4.96	4.08	0.00	0.00	0.22
2.38	5.78	5.74	0.00	0.09	0.51
3.35	0.00	5.73	0.00	0.68	0.00
0.00	5.84	0.00	0.13	0.00	0.38
2.50	7.58	6.84	0.12	0.09	0.39
2.92	6.15	6.69	0.10	0.10	0.27
2.05	8.16	6.20	0.08	0.10	0.33
1.67	6.38	5.27	0.00	0.00	0.14
2.53	3.30	2.03	0.00	0.05	0.16
1.32	6.84	6.81	0.00	0.00	0.19
3.12	7.21	4.84	0.00	0.00	0.20
1.53	4.36	3.85	0.00	0.00	0.38
3.20	7.46	7.01	0.16	0.27	0.28
1.74	5.78	6.91	0.20	0.14	0.34
2.83	6.56	6.44	0.14	0.06	0.38
2.98	6.79	6.82	0.08	0.15	0.30
2.51	5.63	7.08	0.06	0.09	0.27
3.88	3.16	2.28	0.00	0.00	0.19
3.49	4.93	4.07	0.00	0.00	0.14
1.70	4.65	5.37	0.00	0.00	0.12
1.36	4.03	5.10	0.07	0.07	0.42
3.27	5.04	4.43	0.10	0.00	0.28
1.79	4.16	3.66	0.33	0.14	0.35
1.53	4.58	4.17	0.14	0.13	0.29
3.77	2.94	3.05	0.10	0.17	0.34
3.15	3.80	3.32	0.08	0.08	0.33
3.81	4.67	4.47	0.04	0.20	0.26
2.93	3.16	3.23	0.00	0.00	0.23
2.78	2.61	3.49	0.00	0.00	0.38
3.17	3.33	3.28	0.00	0.00	0.59
3.43	4.90	3.78	0.00	0.00	0.28
4.54	5.38	4.55	0.10	0.14	0.95
2.49	0.00	4.67	0.00	0.07	0.00
1.92	4.87	5.24	0.07	0.10	0.25
3.00	3.00	3.77	0.08	0.12	0.46
2.60	3.69	3.56	0.07	0.06	0.20
2.49	4.51	4.54	0.10	0.11	0.52
3.67	5.17	3.40	0.00	0.00	0.54
3.02	4.96	3.62	0.00	0.00	0.18
2.79	6.12	6.28	0.00	0.00	0.29
3.53	7.02	5.64	0.00	0.00	0.25
4.00	4.85	4.28	0.15	0.00	0.66
2.94	3.02	4.58	0.29	0.17	0.60
2.94	5.27	6.14	0.20	0.15	0.30
2.92	6.07	3.78	0.13	0.09	0.40
2.27	6.14	4.92	0.10	0.18	0.23
3.71	5.15	4.80	0.11	0.08	0.33
2.35	4.41	3.86	0.00	0.00	0.26
2.63	4.17	3.89	0.00	0.00	0.39
3.46	3.77	4.07	0.00	0.00	0.46
2.20	6.32	6.13	0.00	0.00	0.34
2.86	4.99	5.74	0.24	0.15	0.57
1.76	4.76	3.82	0.13	0.11	0.31
2.55	3.74	4.87	0.08	0.09	0.45
2.10	6.16	5.92	0.10	0.05	0.27
2.34	7.27	5.70	0.12	0.10	0.29

C22:5n-3_post	C22:6n-3_pre	C22:6n-3_post	total_n-9_pre	total_n-9_post	total_n-6_pre
0.24	0.18	0.14	13.71	12.48	48.92
0.51	0.19	0.43	10.68	9.93	52.60
0.00	0.00	0.11	11.71	13.28	48.63
0.43	0.31	0.32	14.88	16.17	41.63
0.51	0.29	0.40	13.59	14.37	44.33
0.54	0.00	0.38	0.00	11.48	0.00
0.00	0.35	0.00	14.15	0.00	42.14
0.60	0.17	0.37	12.35	11.71	38.63
0.49	0.13	0.29	10.50	12.51	42.40
0.43	0.14	0.18	11.69	10.25	43.10
0.41	0.00	0.38	13.88	12.35	50.04
0.30	0.09	0.13	13.07	13.29	51.98
0.42	0.33	0.40	13.88	14.16	49.10
0.33	0.00	0.00	11.95	11.97	51.15
0.85	0.95	1.00	15.62	13.70	49.21
0.20	0.41	0.14	11.45	13.13	43.97
0.37	0.32	0.40	14.33	15.85	40.60
0.38	0.23	0.21	10.86	10.72	43.26
0.39	0.24	0.28	1.42	12.07	52.69
0.50	0.27	0.25	12.61	12.08	42.30
0.21	0.52	0.41	11.03	12.16	52.20
0.46	0.18	0.46	14.02	9.30	57.32
0.31	0.00	0.21	0.00	13.26	0.00
0.29	0.13	0.13	15.18	13.86	51.28
0.64	0.27	0.35	13.12	14.05	51.37
0.73	0.00	0.81	0.00	11.53	0.00
0.00	0.44	0.00	12.61	0.00	44.38
0.32	0.28	0.25	10.50	9.34	47.42
0.46	0.17	0.30	12.47	10.91	44.81
0.29	0.21	0.11	12.29	12.31	55.96
0.21	0.09	0.12	16.79	18.94	49.30
0.13	0.24	0.40	11.41	11.28	58.80
0.20	0.36	0.22	11.84	13.22	54.25
0.35	0.12	0.27	12.57	12.03	54.93
0.23	0.39	0.35	19.98	21.35	49.78
0.46	0.39	0.31	11.70	10.79	47.60
0.23	0.36	0.26	12.38	12.71	42.79
0.40	0.22	0.24	11.40	11.39	44.35
0.42	0.17	0.22	11.64	11.24	52.94
0.34	0.17	0.16	11.47	12.06	50.52
0.24	0.10	0.20	17.39	15.51	51.91
0.34	0.07	0.20	19.75	13.11	50.18
0.13	0.05	0.00	20.98	17.85	49.36
0.21	0.23	0.18	17.37	22.17	48.86
0.28	0.26	0.25	16.07	16.47	48.45
0.25	0.73	0.10	18.29	25.08	42.30
0.24	0.36	0.22	13.35	22.44	42.14
0.46	0.21	0.38	22.49	12.78	44.78
0.37	0.17	0.28	21.01	18.44	46.69
0.48	0.17	0.48	21.28	14.50	44.33
0.27	0.12	0.20	18.94	16.52	50.70
0.27	0.30	0.21	13.55	13.40	54.21
0.69	0.32	0.38	16.83	15.25	50.81
0.41	0.23	0.33	13.04	14.04	54.53
1.26	1.10	1.24	17.34	15.27	47.16
0.31	0.00	0.39	0.00	17.81	0.00
0.27	0.35	0.14	12.87	20.69	42.44
0.43	0.35	0.28	16.56	18.16	45.16
0.35	0.12	0.15	21.15	23.78	43.26
0.31	0.34	0.22	14.60	19.47	46.75
0.50	0.29	0.17	12.82	12.77	52.68
0.31	0.14	0.23	14.16	12.38	52.57
0.00	0.78	0.00	14.97	14.99	48.90
0.29	0.16	0.28	15.12	13.62	51.38
0.71	0.46	0.35	12.93	13.13	47.14
0.48	0.63	0.30	10.61	11.74	45.24
0.37	0.42	0.33	11.87	11.58	39.26
0.47	0.20	0.22	11.91	10.78	44.86
0.37	0.09	0.06	12.75	11.56	48.53
0.46	0.17	0.31	11.21	11.05	48.82
0.35	0.15	0.32	12.77	12.61	52.22
0.37	0.39	0.45	12.50	13.08	50.45
0.36	0.36	0.24	10.97	11.67	56.81
0.36	0.24	0.24	13.90	14.07	48.39
0.41	0.75	0.39	14.17	14.58	42.66
0.25	0.42	0.29	12.94	14.84	43.40
0.39	0.25	0.30	11.90	12.28	45.62
0.33	0.17	0.21	12.47	12.42	44.81
0.30	0.13	0.16	12.27	11.87	43.44

total_n-6_post	total_n-3_pre	total_n-3_post	total_SFA_pre	total_SFA_post	total_MUFA_pre
49.25	6.39	10.58	30.29	27.19	14.41
54.69	10.11	4.87	24.64	28.77	12.64
52.50	14.15	7.76	24.97	25.98	12.24
41.22	11.75	10.72	30.60	30.55	16.02
45.81	11.40	8.38	29.62	30.61	14.66
48.96	0.00	9.08	0.00	27.84	0.00
0.00	6.05	0.00	35.18	0.00	16.64
43.91	12.48	8.14	34.76	34.18	14.12
47.60	13.82	7.59	30.82	30.22	12.96
45.83	13.10	13.25	30.04	28.75	13.75
53.16	7.24	6.82	27.72	27.06	15.00
56.46	8.70	3.95	25.70	25.64	13.63
45.44	8.65	12.36	27.40	26.83	14.86
53.95	10.04	7.38	25.94	25.83	12.87
49.80	6.86	7.63	27.50	28.08	16.43
44.61	10.21	11.97	32.14	27.92	13.68
39.78	8.26	7.43	33.40	34.19	17.73
47.14	9.48	8.17	34.22	32.06	13.04
47.22	9.31	9.47	34.25	28.93	3.75
47.34	12.71	10.64	30.58	27.87	14.40
54.77	6.24	6.19	28.67	25.15	12.89
56.45	7.37	4.20	19.83	28.24	15.48
53.96	0.00	6.24	0.00	24.79	0.00
53.13	5.32	4.50	25.81	26.26	17.59
49.00	6.56	6.82	26.89	27.76	15.18
51.16	0.00	7.95	0.00	26.91	0.00
0.00	6.80	0.00	33.57	0.00	15.25
47.17	8.37	7.50	31.64	34.72	12.57
51.51	6.69	7.54	33.08	28.01	15.42
50.84	8.78	6.70	20.47	28.28	14.79
46.80	6.61	5.60	25.18	26.35	18.91
58.39	3.70	2.62	24.22	25.89	13.28
53.18	7.40	7.23	24.90	24.70	13.45
57.11	7.53	5.46	23.49	23.88	14.05
49.27	5.12	4.44	22.95	23.15	22.14
50.85	8.30	8.05	30.27	28.26	13.83
47.30	6.68	7.53	35.49	30.10	15.04
51.25	7.30	7.14	34.20	28.04	14.15
53.04	7.34	7.60	26.51	26.45	13.21
50.66	6.13	7.67	30.02	27.76	13.33
55.69	3.46	2.73	25.79	24.57	18.85
56.61	5.14	4.61	23.44	24.19	21.24
49.41	4.83	5.50	23.56	25.71	22.25
38.45	4.75	5.56	26.96	31.37	19.43
48.83	5.69	4.96	27.77	28.13	18.09
41.04	5.55	4.16	31.91	27.82	20.23
42.93	5.37	4.76	36.25	27.97	16.24
50.61	3.59	4.05	27.49	30.67	24.15
47.76	4.38	4.05	26.11	28.17	22.82
50.35	5.13	5.63	27.52	27.82	23.01
52.24	3.51	3.71	25.28	25.72	20.51
54.47	3.29	3.97	27.01	26.18	15.49
53.49	4.24	4.34	26.45	25.34	18.51
52.91	5.41	4.52	25.61	26.66	14.46
47.48	7.54	7.19	25.66	27.63	19.64
47.98	0.00	5.44	0.00	26.90	0.00
42.95	5.54	5.75	36.65	28.58	15.37
47.14	3.89	4.60	32.43	28.24	18.51
46.47	4.09	4.12	28.58	23.70	24.07
43.39	5.47	5.18	31.10	29.65	16.68
51.99	6.00	4.06	27.18	29.54	14.14
53.07	5.29	4.16	26.02	28.50	16.12
51.44	7.19	6.28	28.31	26.56	15.60
52.49	7.43	6.22	24.48	26.05	16.71
47.46	6.13	5.34	31.16	31.03	15.57
50.12	4.55	5.54	37.04	30.13	13.18
47.86	6.19	6.99	40.23	31.63	14.32
49.36	6.80	4.56	33.99	33.35	14.34
48.14	6.55	5.53	29.82	32.58	15.10
52.75	5.76	5.65	32.44	28.84	12.98
54.70	4.82	4.53	27.82	26.01	15.14
51.65	4.94	4.71	29.75	28.54	14.86
55.29	4.59	4.67	25.88	26.88	12.72
47.81	6.90	6.72	28.49	28.96	16.21
45.46	6.56	6.69	33.89	31.11	16.89
43.49	5.62	4.48	35.33	34.81	15.66
47.07	4.52	5.65	35.84	32.38	14.02
47.79	6.69	6.51	33.08	30.55	15.42
50.01	7.82	6.27	33.67	29.68	15.06

total_MUFA_post	total_PUFA_pre	total_PUFA_post	n-6:n-3_pre	n-6:n-3_post	M:S_pre
12.97	55.31	59.83	7.66	4.65	0.48
11.66	62.71	59.56	5.20	11.23	0.51
13.76	62.78	60.26	3.44	6.76	0.49
17.51	53.38	51.94	3.54	3.84	0.52
15.19	55.73	54.20	3.89	5.47	0.49
14.11	0.00	58.04	0.00	5.39	0.00
0.00	48.19	0.00	6.96	0.00	0.47
13.76	51.12	52.06	3.09	5.39	0.41
14.59	56.22	55.19	3.07	6.27	0.42
12.17	56.20	59.08	3.29	3.46	0.46
12.97	57.28	59.97	6.91	7.80	0.54
13.95	60.68	60.40	5.97	14.31	0.53
15.37	57.75	57.80	5.68	3.68	0.54
12.84	61.19	61.34	5.10	7.31	0.50
14.49	56.07	57.43	7.17	6.53	0.60
15.49	54.18	56.58	4.31	3.73	0.43
18.60	48.87	47.21	4.91	5.35	0.53
12.63	52.74	55.30	4.57	5.77	0.38
14.39	62.00	56.68	5.66	4.99	0.11
14.14	55.01	57.98	3.33	4.45	0.47
13.88	58.44	60.97	8.37	8.84	0.45
11.11	64.69	60.65	7.78	13.45	0.78
15.01	0.00	60.19	0.00	8.65	0.00
16.11	56.60	57.63	9.64	11.80	0.68
16.43	57.93	55.81	7.83	7.19	0.56
13.98	0.00	59.11	0.00	6.44	0.00
0.00	51.18	0.00	6.53	0.00	0.45
10.61	55.79	54.67	5.67	6.29	0.40
12.94	51.50	59.05	6.70	6.83	0.47
14.19	64.74	57.54	6.37	7.59	0.72
21.25	55.92	52.40	7.45	8.36	0.75
13.10	62.50	61.01	15.91	22.27	0.55
14.89	61.65	60.41	7.33	7.35	0.54
13.54	62.46	62.57	7.29	10.45	0.60
23.14	54.90	53.70	9.72	11.11	0.96
12.83	55.90	58.91	5.74	6.31	0.46
15.07	49.47	54.83	6.41	6.28	0.42
13.57	51.65	58.39	6.07	7.18	0.41
12.91	60.28	60.64	7.21	6.98	0.50
13.90	56.65	58.33	8.24	6.60	0.44
17.00	55.36	58.42	15.01	20.40	0.73
14.59	55.32	61.22	9.76	12.28	0.91
19.38	54.19	54.91	10.23	8.98	0.94
24.62	53.61	44.01	10.28	6.91	0.72
18.08	54.14	53.79	8.51	9.84	0.65
26.98	47.86	45.20	7.62	9.87	0.63
24.33	47.51	47.69	7.85	9.02	0.45
14.67	48.37	54.66	12.48	12.50	0.88
20.02	51.07	51.81	10.66	11.79	0.87
16.20	49.47	55.98	8.64	8.94	0.84
18.33	54.21	55.95	14.45	14.09	0.81
15.38	57.50	58.44	16.48	13.72	0.57
16.83	55.04	57.83	11.98	12.33	0.70
15.90	59.94	57.44	10.09	11.69	0.56
17.70	54.70	54.67	6.26	6.60	0.77
19.68	0.00	53.42	0.00	8.81	0.00
22.72	47.98	48.70	7.67	7.47	0.42
20.02	49.06	51.74	11.60	10.25	0.57
25.70	47.35	50.59	10.58	11.28	0.84
21.78	52.22	48.57	8.54	8.38	0.54
14.41	58.68	56.05	8.79	12.81	0.52
14.27	57.86	57.23	9.93	12.76	0.62
15.73	56.09	57.72	6.80	8.19	0.55
15.24	58.81	58.71	6.91	8.44	0.68
16.16	53.27	52.80	7.69	8.88	0.50
14.21	49.79	55.66	9.95	9.05	0.36
13.52	45.45	54.85	6.34	6.84	0.36
12.73	51.67	53.92	6.59	10.81	0.42
13.75	55.08	53.67	7.41	8.71	0.51
12.75	54.58	58.41	8.48	9.33	0.40
14.76	57.05	59.22	10.83	12.08	0.54
15.10	55.39	56.36	10.21	10.95	0.50
13.16	61.40	59.96	12.38	11.85	0.49
16.51	55.30	54.53	7.01	7.11	0.57
16.75	49.22	52.15	6.50	6.79	0.50
17.23	49.01	47.97	7.73	9.71	0.44
14.90	50.14	52.71	10.10	8.34	0.39
15.15	51.50	54.30	6.70	7.35	0.47
14.04	51.26	56.28	5.56	7.98	0.45

M:S_post	P:S_pre	P:S_post
0.48	1.83	2.20
0.41	2.54	2.07
0.53	2.51	2.32
0.57	1.74	1.70
0.50	1.88	1.77
0.51	0.00	2.08
0.00	1.37	0.00
0.40	1.47	1.52
0.48	1.82	1.83
0.42	1.87	2.06
0.48	2.07	2.22
0.54	2.36	2.36
0.57	2.11	2.15
0.50	2.36	2.38
0.52	2.04	2.05
0.55	1.69	2.03
0.54	1.46	1.38
0.39	1.54	1.72
0.50	1.81	1.96
0.51	1.80	2.08
0.55	2.04	2.42
0.39	3.26	2.15
0.61	0.00	2.43
0.61	2.19	2.19
0.59	2.15	2.01
0.52	0.00	2.20
0.00	1.52	0.00
0.31	1.76	1.57
0.46	1.56	2.11
0.50	3.16	2.03
0.81	2.22	1.99
0.51	2.58	2.36
0.60	2.48	2.45
0.57	2.66	2.62
1.00	2.39	2.32
0.45	1.85	2.08
0.50	1.39	1.82
0.48	1.51	2.08
0.49	2.27	2.29
0.50	1.89	2.10
0.69	2.15	2.38
0.60	2.36	2.53
0.75	2.30	2.14
0.79	1.99	1.40
0.64	1.95	1.91
0.97	1.50	1.62
0.87	1.31	1.70
0.48	1.76	1.78
0.71	1.96	1.84
0.58	1.80	2.01
0.71	2.14	2.17
0.59	2.13	2.23
0.66	2.08	2.28
0.60	2.34	2.15
0.64	2.13	1.98
0.73	0.00	1.99
0.79	1.31	1.70
0.71	1.51	1.83
1.08	1.66	2.13
0.73	1.68	1.64
0.49	2.16	1.90
0.50	2.22	2.01
0.59	1.98	2.17
0.59	2.40	2.25
0.52	1.71	1.70
0.47	1.34	1.85
0.43	1.13	1.73
0.38	1.52	1.62
0.42	1.85	1.65
0.44	1.68	2.02
0.57	2.05	2.28
0.53	1.86	1.98
0.49	2.37	2.23
0.57	1.94	1.88
0.54	1.45	1.68
0.49	1.39	1.38
0.46	1.40	1.63
0.50	1.56	1.78
0.47	1.52	1.90
